# Pyran Rings Containing Polyketides from *Penicillium raistrickii*

**DOI:** 10.3390/md15010002

**Published:** 2016-12-23

**Authors:** Li-Ying Ma, De-Sheng Liu, De-Guo Li, Yu-Ling Huang, Hui-Hui Kang, Chun-Hua Wang, Wei-Zhong Liu

**Affiliations:** 1 College of Pharmacy, Binzhou Medical College, Yantai 264003, China; maliyingbz@163.com (L.-Y.M.); desheng_liu@sina.com (D.-S.L.); huangyuling1979@163.com (Y.-L.H.); kanghuihui_1993@126.com (H.-H.K.); chhwang77@163.com (C.-H.W.); 2 The Hospital of Luzhong Mining Co., Ltd., Laiwu 271113, China; deguoli1983@sina.com

**Keywords:** *Penicillium raistrickii*, α-pyrone, isocoumarin, polyketides, saline soil-derived fungus

## Abstract

Five new pyran rings containing polyketides, penicipyrans A–E (**1**–**5**), together with the known pestapyrone A (**6**), were isolated from the saline soil-derived *Penicillium raistrickii*. Their structures were determined by interpretation of NMR and HRESIMS data. The absolute configurations of compounds **4** and **5** were established by the modified Mosher’s method and single-crystal X-ray diffraction analysis, respectively. These compounds possessed high structural diversity including two α-pyrones (**1**, **2**), three isocoumarins (**3**, **4**, **6**), and one dihydropyran derivative (**5**). Among them, Compound **5** exhibited cytotoxicity against HL-60 and K562 cell lines with IC_50_ values of 4.4 and 8.5 μM, respectively.

## 1. Introduction

Natural products are intriguing and a promising source of anticancer drugs [1]. Those produced by organisms living in special environments, such as high pH, high/low temperature, high salty, etc., represent a group of compounds possessing unique chemical scaffolds that are important for finding new drug leads [2,3,4,5]. The saline soil broadly distributes near the seaside, which features a complex ecosystem with high salt and pH, and low nutrients, due to the interaction of land and sea [6]. To adapt to special environments, fungal strains inhabiting saline niches represent an excellent biosynthesizer with great potential to produce structurally diverse and biologically important secondary metabolites.

**Figure 1:**
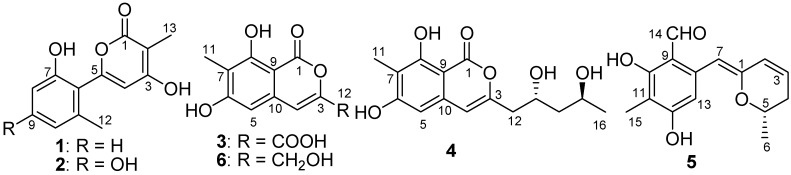
Structures of Compounds **1**–**6**.

In the course of our ongoing search for bioactive natural products from saline soil-derived fungi [7,8], a *P. raistrickii* strain isolated from saline soil collected from the coast of Bohai Bay in Zhanhua, China, was selected for further study because of the potent anti-proliferative activity of its EtOAc extract. In the previous study, a series of new spiroketals with cytotoxcity were obtained from the same strain [9,10]. Encouraged by the findings, we carried out a study that lead to the isolation of five new metabolites with pyran moiety, including two α-pyrones (**1**, **2**), two isocoumarins (**3**, **4**), and one dihydropyran derivative (**5**), called penicipyran A–E (**1**–**5**) (Figure 1) [11]. Herein, we report the isolation, structure elucidation, and cytotoxicity of these compounds.

## 2. Results

**Figure 2:**
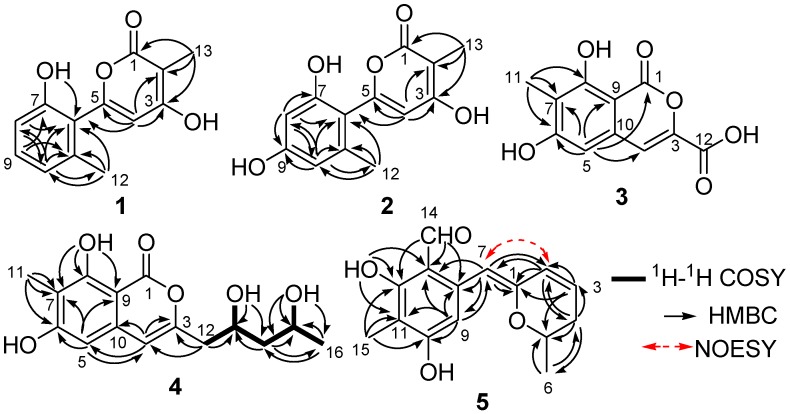
Selected ^1^H-^1^H COSY, HMBC, and key NOESY correlations in Compounds **1**–**5**.

Penicipyran A (**1**) was obtained as a colorless needle. HRESIMS produced an ion peak at *m*/*z* 231.0659 [M − H]^−^, indicating a molecular formula of C_13_H_12_O_4_. The ^1^H NMR data (Table 1) showed the presence of two methyl groups (δ_H_ 2.15, 1.83, 3H, each), three vicinal aromatic protons of an ABC spin system at δ_H_ 7.16 (1H, dd, *J* = 8.2, 7.5 Hz), 6.76 (1H, d, *J* = 8.2 Hz), and 6.73 (1H, d, *J* = 7.5 Hz), an isolated olefinic proton at δ_H_ 6.17, and two phenolic hydroxyl protons at δ_H_ 11.21 and 9.73. The ^13^C NMR spectrum displayed 13 carbon signals. Eleven of them were aromatic or olefinic carbons, including a carbonyl and three oxygenated ones, and two of them were methyl carbons. An α-pyrone moiety was deduced from the HMBC correlations from H-13 to C-1, C-2, and C-3, and from H-4 to C-2, C-3, and C-5, as well as the chemical shifts of C-1–C-5. The IR absorptions at 1652 and 1562 cm^−1^ and UV maximum absorption at 291 nm also supported the presence of the α-pyrone moiety [12]. The HMBC correlation (Figure 2) from H-4 to C-6 suggested a linkage between the 1,2,3-trisubstituted phenyl ring and the α-pyrone moiety from C-5 to C-6. To satisfy the molecular formula, two hydroxyl groups were assigned to C-3 and C-7, respectively, which was confirmed by their chemical shifts (Table 1), as well as the HMBC correlations (Figure 2). Thus, the structure of **1** was established.

Penicipyran B (**2**) was isolated as colorless needles. Its molecular formula was assigned to be C_13_H_12_O_5_ on the basis of negative HRESIMS ion at *m*/*z* 247.0604 [M − H]^−^, which was one more oxygen atom than that of **1**. The IR and UV spectra of Compounds **1** and **2** were almost identical, indicating they were structural analogues. The ^13^C NMR spectrum of **2** also showed 13 carbon signals (Table 1), which was highly similar to those of **1**, except for the presence of an oxygenated aromatic quaternary carbon instead of an aromatic methine in **1**. Those findings suggested **2** was a hydroxylated derivative of **1**. The additional hydroxyl in **2** was assigned at C-9, supported by the observation of two isolated aromatic hydrogen signals at δ_H_ 6.34 and 6.30, and the chemical shift of C-9. The structure was further confirmed by the HMBC spectrum (Figure 2).

Penicipyran C (**3**) was acquired as a pale yellowish amorphous powder. Its molecular formula C_11_H_8_O_6_ was assigned based upon the HRESIMS ion at *m/z* 235.0246 [M − H]^−^. The UV spectrum (absorption peaks at 340, 330, 304, and 254 nm) was similar to that of 6,8-dihydroxyisocoumarin-3-carboxylic acid [13]. In the IR spectrum, the broad absorption peak at 3600–2500 cm^−1^ indicated the presence of a carboxyl group. The ^1^H NMR spectrum displayed two isolated aromatic hydrogen signals at δ_H_ 7.21 (1H, s) and 6.69 (1H, s), and one aromatic methyl at δ_H_ 2.04 (3H, s). The ^13^C NMR spectrum showed ten sp^2^ carbon signals and one aliphatic carbon signal (Table 1). Carefully comparing the data of **3** with those of 6,8-dihydroxyisocoumarin-3-carboxylic acid and pestapyrone A (**6**) [14], likewise isolated in this study, revealed that the presence of a carboxylic acid group in **3** in place of a hydroxymethyl group in **6**. Finally, the HSQC and HMBC spectra were used to confirm the structure of **3**.

Penicipyran D (**4**), a colorless needle, was assigned the molecular formula C_15_H_18_O_6_ based on the HRESIMS ion at *m/z* 293.1015 [M − H]^−^. The UV (absorption peaks at 333, 280, 247, and 240 nm) and IR (absorptions at 1672, 1621, 1582 and 1510 cm^−1^) spectra of **4** were almost the same as those of **6** and peneciraistin D [10], which suggested they possessed the same isocoumarin moiety. The NMR data (Table 2) also strongly supported that Compound **4** contained the same isocoumarin moiety as **6**, except additional signals for two hydroxyls at δ_H_ 4.72 (1H, d, *J* = 5.6 Hz) and 4.38 (1H, d, *J* = 4.9 Hz), two oxygenated methines, two methylenes, and a methyl group were observed in the spectra. The ^1^H-^1^H COSY data (Figure 2) established the presence of a 2,4-dihydroxyl pentyl moiety. Finally, the HMBC correlations from H-12 to C-3 and C-4 indicated the connection between pentyl moiety and isocoumarin moiety, which established the planar structure of **4**.

**Figure 3:**
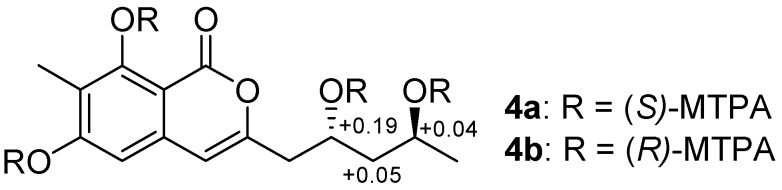
Δδ*_S-R_* values of (*R*)- and (*S*)-MTPA esters of **4**.

The coupling constants of H_a_-14 [δ_H_ 1.61 (1H, ddd, *J* = 14.2, 8.8, 3.3 Hz)] and H_b_-14 [δ_H_ 1.56 (1H, ddd, *J* = 14.2, 9.0, 3.4 Hz)] in the ^1^H NMR spectrum (MeOH-*d*_6_, 500 MHz) (Figure S28) revealed two hydroxyls were on the opposite sides [15]. In order to determine its absolute configuration, **4** was treated with (*R*)- and (*S*)-MTPA chlorides to produce its (*S*)- and (*R*)-MTPA esters (**4a** and **4b**), respectively. According to the distributions of the Δδ*_S_*_-*R*_ values (Figure 3) for 1,3-anti-diol model as reported [16,17,18], the positive Δδ*_S_*_-*R*_ values of C-13 and C-15 suggested their absolute configurations to be 13*R* and 15*S*.

**Figure 4:**
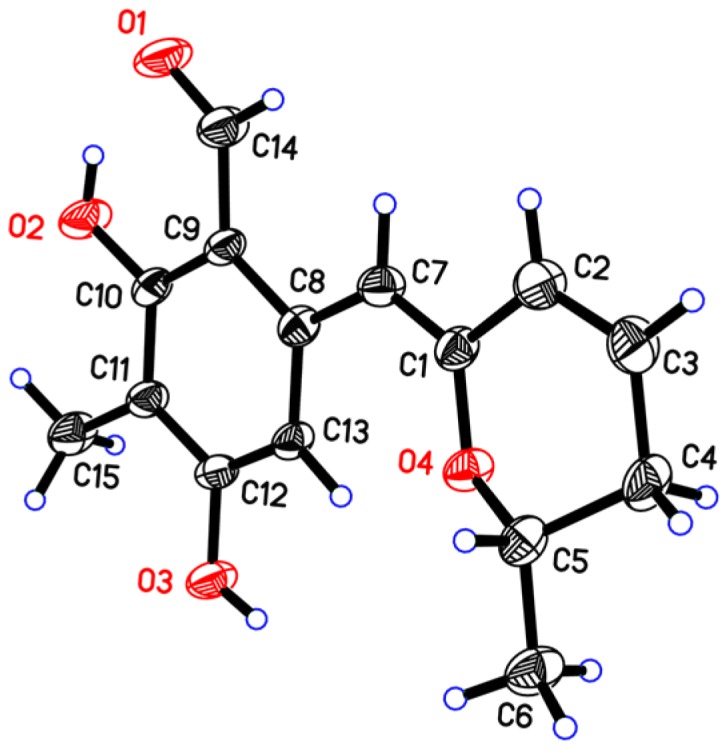
X-ray structure of penicipyran E (**5**).

Penicipyran E (**5**) was obtained as yellow needles. Its molecular formula C_15_H_16_O_4_ was established by HRESIMS at *m*/*z* 259.0965 [M − H]^−^, which was in agreement with the ^1^H and ^13^C NMR data. An aldehyde, two phenolic hydroxyls, an aromatic methyl and an isolated aromatic hydrogen proton were observed in the ^1^H and ^13^C NMR spectra (Table 2). The 1D NMR data were similar to those of **4**, except two major differences were observed: (1) the observation of an aldehyde group (δ_C_ 193.8, δ_H_ 10.08) in **5** instead of an ester carbonyl in **4**; (2) a -CH_2_-CHOH- moiety in **4** was replaced by a double bond (δ_C_ 124.8, δ_H_ 6.24; δ_C_ 127.9, δ_H_ 6.09) in **5**. These findings indicated that there was a different polyketide cyclization pattern for Compound **5**, forming a hydro-pyran ring at the terminus instead of the benzoenol lactone in **4**. The structure of **5** was further conformed by comprehensive 2D NMR analyses (Figure 2) and single crystal X-ray diffraction analyses (Figure 4), which finally established the absolute configuration of **5** as 1*Z*, 6*S*.

Compounds **1**–**6** were tested for their cytotoxicity against a panel of cancer cell lines (A549, HL-60, and K562) using previously described methods [8,10], with doxorubicin as a positive control (IC_50_s: 0.42, 0.15, and 0.33 μM, respectively). Compounds **5** showed cytotoxicity against HL-60 and K562 cell lines with IC_50_ values of 4.4 and 8.5 μM, respectively, while the other compounds were inactive (IC_50_ > 20 μM).

## 3. Materials and Methods

### 3.1. General Experimental Procedures

Melting point (mp) data were obtained with an XRC-1 micro-melting point apparatus (Sichuan University Scientific Instrument Factory, Chengdu, China). Optical rotations were determined on an Autopol V Plus digital polarimeter (Rudolph Research Analytical, Hackettstown, NJ, USA). UV spectra were recorded on a TU-1091 spectrophotometer (Beijing Purkinje General Instrument Co., Ltd., Beijing, China). CD spectra were acquired with a Chirascan spectropolarimeter (Applied Photophysics Ltd., Leatherhead, UK). IR spectra were collected on a Nicolet 6700 spectrophotometer (Thermo Scientific, Waltham, MA, USA) using an attenuated total reflectance (ATR) method. NMR spectra were recorded on a Bruker Avance 400 (Bruker Biospin Group, Karlsruhe, Germany) or Bruker AVIII 500 spectrometer (Bruker Biospin Group, Karlsruhe, Germany) with TMS as the internal standard. Crystal structure determination was carried out on a Bruker Smart 1000 CCD X-ray diffractometer (Bruker Biospin Group, Karlstuhe, Germany). HRESIMS was measured on a 1200RRLC-6520 Accurate-Mass Q-TOF LC/MS mass spectrometer (Agilent Technologies, Ltd., Palo Alto, CA, USA). Semipreparative HPLC was performed on a SHIMADZU LC-6AD Liquid Chromatography (Shimadzu Corporation, Kyoto, Japan) with an SPD-20A Detector using an ODS column (HyperClone 5 μ ODS (C_18_) 120 Å, 250 mm × 10 mm, Phenomenex, 4 mL/min). Sephadex LH-20 (Ge Healthcare Bio-Sciences AB, Uppsala, Sweden).

### 3.2. Fungal Material

The working strain, *P. raistrickii* (Genbank accession No. HQ717799), was isolated from the saline soil collected from the coast of Bohai Bay in Zhanhua, Shandong Province of China, in August 2008. The fungus was identified on the basis of sequence analysis of the ITS region of the rDNA. It was deposited at Department of Chemistry, Binzhou Medical College, Yantai. The fermentation and extraction of *P. raistrickii* were described in a previous article [10].

### 3.3. Extraction and Isolation

The extract (42 g) of *P. raistrickii* was subjected to a silica gel (200–300 mesh) column eluted with a stepwise gradient of petroleum ether/chloroform (2:1, 1:1, 1:2, and 0:1, *v*/*v*), and chloroform/methanol mixtures (100:1, 50:1, 20:1, 10:1, and 0:1, *v*/*v*) to yield eight fractions (Fractions 1–8). Fraction 6 (3.8 g) was passed through an ODS column (25–40 μm, Merck, Darmstadt, Germany) eluting with a H_2_O/CH_3_OH gradient to yield eighteen subfractions (Subfractions 6-1–6-18). Subfraction 6-5 (0.2 g) was purified by semipreparative HPLC on an ODS column using MeOH/H_2_O (4:6, *v*/*v*; 1 L of water was added to 2 mL of trifluoroacetic acid; 4 mL/min) as the eluting solvent to yield **1** (13 mg, *t*_R_ 22.1 min) and **6** (26 mg, *t*_R_ 25.8 min). Subfraction 6-2 (0.1 g) was subjected to semipreparative HPLC (MeOH/H_2_O, 3:7, *v*/*v*; 1 L of water contained 2 mL of trifluoroacetic acid; 4 mL/min) to obtain **2** (21 mg, *t*_R_ 24.6 min). Compounds **3** (7 mg) and **4** (22 mg) were obtained from Fraction 4 (1.1 g) through Sephadex LH-20 eluting with methanol. Fraction 2 (6.3 g) was fractionated using an ODS column eluting with a H_2_O/CH_3_OH gradient to yield fourteen subfractions (Subfractions 2-1–2-14). Subfraction 2-8 (0.3 g) was purified on Sephadex LH-20 using methanol as eluant to yield Compound **5** (16 mg).

Penicipyran A (**1**): colorless needles; mp 204–206 °C (MeOH); UV (MeOH) λ_max_ (log ε) 291 (3.90), 214 (4.37) nm; IR (ATR) ν_max_ 2935, 2619, 1652, 1612, 1562, 1464, 1402, 1375, 1222, 1134, 1063, 960, 910, 878, 828, 779, 752, 738, 701 cm^−1^; ^1^H and ^13^C NMR data, see Table 1; HRESIMS *m*/*z*: 231.0659 [M − H]^−^ (calcd. for C_13_H_11_O_4_, 231.0652).

Penicipyran B (**2**): colorless needles; mp 245–248 °C (MeOH); UV (MeOH) λ_max_ (log ε) 299 (3.67), 219 (4.04); IR (ATR) ν_max_ 3335, 2931, 2598, 1699, 1646, 1606, 1549, 1471, 1404, 1374, 1340, 1274, 1250, 1223, 1120, 1055, 990, 949, 904, 881, 830, 753, 733, 706 cm^−1^; ^1^H and ^13^C NMR data, see Table 1; HRESIMS *m*/*z* 247.0604 [M − H]^−^ (calcd. for C_13_H_11_O_5_, 247.0601).

Penicipyran C (**3**): pale yellowish amorphous power (MeOH); UV (MeOH) λ_max_ (log ε) 340 (3.80), 330 (3.77), 304 (3.79), 254 (4.50), 205 (4.04) nm; IR (ATR) ν_max_ 3600–2500 (broad), 1729, 1662, 1614, 1515, 1435, 1392, 1347, 1247, 1198, 1149, 1123, 1087, 977, 889, 827, 788, 760 cm^−1^; ^1^H and ^13^C NMR data, see Table 1; HRESIMS *m*/*z* 235.0246 [M − H]^−^ (calcd. for C_11_H_7_O_6_, 235.0248).

Penicipyran D (**4**): colorless needles; mp 222–224 °C (MeOH); ${\left[\alpha \right]}_{D}^{20}$ −21.9 (*c*, 0.05, CH_3_OH). UV (MeOH) λ_max_ (log ε) 333 (3.39), 280 (3.46), 247 (4.28), 240 (4.23), 201 (3.98) nm; CD (*c*, 0.5 mg/mL, MeOH) λ_max_ (Δε) 336 (−3.93), 289 (−7.65) nm; IR (ATR) ν_max_ 3126, 2966, 1672, 1621, 1585, 1510, 1462, 1439, 1348, 1259, 1206, 1176, 1152, 1104, 1058, 986, 834, 790, 715, 693 cm^−1^; ^1^H NMR (MeOH-*d*_4_, 500 MHz) δ_H_ 6.35 (2H, s, H-4 and 5), 4.23 (1H, m, H-13), 4.03 (1H, m, H-15), 2.66 (1H, dd, *J* = 14.5, 5.0 Hz, H_a_-12), 2.59 (1H, dd, *J* = 14.5, 8.1Hz, H_b_-12), 2.09 (3H, s, H-11), 1.61 (1H, ddd, *J* = 14.2, 8.8, 3.3 Hz, H_a_-14), 1.56 (1H, ddd, *J* = 14.2, 9.0, 3.4 Hz, H_a_-14), 1.21 (3H, d, *J* = 6.3 Hz, H-16); ^1^H (400 MHz) and ^13^C NMR (100 MHz) data, see Table 2; HRESIMS *m*/*z* 293.1015 [M − H]^−^ (calcd. for C_15_H_17_O_6_, 293.1020).

Penicipyran E (**5**): yellow needles; mp 168–170 °C (MeOH); ${\left[\alpha \right]}_{D}^{20}$ +94.3 (*c*, 0.14, CH_3_OH). UV (MeOH) λ_max_ (log ε) 371 (3.88), 322 (4.14), 272 (4.45), 215 (4.05); CD (*c*, 0.5 mg/mL, MeOH) λ_max_ (Δε): 330 (+4.78), 289 (+1.76), 270 (−12.78), 212 (+15.75) nm; IR (ATR) ν_max_ 3374, 2917, 2849, 1630, 1599, 1464, 1429, 1397, 1335, 1246, 1122, 1111, 1075, 1053, 962, 851, 801, 768, 750, 727, 674 cm^−1^; ^1^H and ^13^C NMR data, see Table 2; HRESIMS *m*/*z* 259.0965 [M − H]^−^(calcd. for C_15_H_15_O_4_, 259.0965).

### 3.4. X-ray Crystal Data for Penicipyran E *(**5**)*

Penicipyran E was obtained as a yellow needle from MeOH: molecular formula C_15_H_16_O_4_; *M*_r_ = 260.28, triclinic, space group *P*1, *a* = 8.5057(5) Å, *b* = 8.5593(5) Å, *c* = 9.4977(5) Å, α = 102.001(3)°, β = 101.812(3)°, γ = 97.7165(17)°, *V* = 650.64(6) Å^3^, *Z* = 2, *T* = 293 (2) K, μ (Cu Kα) = 0.792 mm^−1^, *D*_calc_ = 1.329 g/cm^3^, *F* (000) = 276, 4030 reflections measured (9.80 ≤ 2Θ ≤ 132.00), 2600 unique (*R*_int_ = 0.0129) which were used in all calculations. The final *R*_1_ was 0.0427 [*I* ≥ 2σ (I)] and *wR*_2_ was 0.1318 (all data). The crystal structure of Compound **5** was solved by direct method SHELXS-97 and expanded using difference Fourier techniques, refined by the program SHLXL-97 and full-matrix least-squares calculations. Crystallographic data for the structure of penicipyran E have been deposited with the Cambridge Crystallographic Data Centre as supplementary publication CCDC 1512473. These data can be obtained free of charge from the Cambridge Crystallographic Data Centre via www.ccdc.cam.ac.uk/data_request/cif.

### 3.5. Preparation of (R)- and (S)-MTPA Esters of Penicipyran D *(**4**)*

As our previously reported method [10], (*R*)-MTPA chloride (20 μL) and DMAP (50 μg) were added to a solution of **4** (2.0 mg) in pyridine (0.5 mL). The mixture was stirred at 28 °C for 12 h. After the addition of water (1 mL) and extraction with EtOAc, the extract was evaporated and the residue was subjected to HPLC on an ODS column (MeOH/H_2_O, 90:10, 4 mL/min) to obtain the (*S*)-MTPA ester (**4a**). The same procedure was used to prepare the (*R*)-MTPA ester (**4b**) with (*S*)-MTPA chloride. ^1^H NMR (CDCl_3_, 400 MHz) data of pentyl chain in **4a**: δ_H_ 5.33 (1H, m, H-13), 5.10 (1H, m, H-15), 2.71 (2H, m, H-12), 1.98 (2H, m, H-14), 1.28 (3H, *J* = 5.2 Hz, H-16); ^1^H NMR (CDCl_3_, 400 MHz) data of pentyl chain in **4b**: δ_H_ 5.14 (1H, m, H-13), 5.06 (1H, m, H-15), 2.72 (2H, m, H-12), 1.93 (2H, m, H-14), 1.32 (3H, d, *J* = 5.2 Hz, H-16).

## 4. Conclusions

Penicipyrans (**1**–**6**) are members of a large family of pyran-containing natural products, including flavones, coumarins, isocoumarins, pyrones, xanthones, spiroketals, and others. Those compounds have a wide range of biological activities, including antitumor [19,20,21], antimicrobial [22], anti-inflammatory [23], and protective activities of the central nervous system (CNS) [24,25], which provide attractive chemicals for drug discovery [26]. In this research, five new pyran-bearing compounds, penicipyran A–E, were isolated from the extract of *P. raistrichii*. Penicipyran E (**5**) showed cytotoxicity against HL-60 and K562 cell lines. This finding provides a series of pyran-bearing chemicals that may be applied for the analyses of structure activity relationships of analogues in the future.

## Figures and Tables

**Table 1:** ^1^H and ^13^CNMR data for **1** (DMSO-*d*_6_), **2** (acetone-*d*_6_) and **3** (DMSO-*d*_6_).

Position	1 ^a^		2 ^b^		3 ^a^	
	δ_C_	δ_H_ (*J* in Hz)	δ_C_	δ_H_ (*J* in Hz)	δ_C_	δ_H_ (*J* in Hz)
1	165.3, C		166.2, C		166.3, C	
2	97.5, C		98.3, C			
3	164.5, C		164.7, C		149.2, C	
4	103.6, CH	6.17, s	104.1, CH	6.28, s	108.4, CH	7.21, s
5	155.7, C		156.6, C		103.4, CH	6.69, s
6	120.2, C		112.5, C		163.8, C	
7	155.4, C		156.7, C		111.0, C	
8	113.3, CH	6.76, d (8.2)	100.4, CH	6.34, s	159.9, C	
9	130.5, CH	7.16, dd (8.2, 7.5)	159.3, C		98.7, C	
10	120.6, CH	6.73, d (7.5)	109.0, CH	6.30, s	135.7, C	
11	137.8, C		139.6, C		8.1, CH_3_	2.04, s
12	19.4, CH_3_	2.15, s	19.4, CH_3_	2.17, s	162.1, C	
13	8.5, CH_3_	1.83, s	7.8, CH_3_	1.92, s		
OH-3		11.21, s				
OH-7		9.73, s				
OH-8						11.39, br s

^a^ The ^1^H (400 M Hz) and ^13^C (100 M Hz); ^b^ The ^1^H (500 M Hz) and ^13^C (125 M Hz).

**Table 2:** ^1^H and ^13^C NMR data for **4** (DMSO-*d*_6_) and **5** (acetone-*d*_6_).

Position	4 ^a^			5 ^b^
	δ_C_	δ_H_ (*J* in Hz)	δ_C_	δ_H_ (*J* in Hz)
1	166.3, C		152.5, C	
2			124.8, CH	6.24, dd (9.7, 2.5)
3	154.6, C		127.9, CH	6.09, ddd (9.7, 6.5, 2.6)
4	105.3, CH	6.47, s	31.4, CH_2_	2.34, ddd (18.0, 6.1, 3.5); 2.16, ddt (18.0, 10.3, 2.5)
5	101.5, CH	6.44, s	71.6, CH	4.11, m
6	163.5, C		20.3, CH_3_	1.34, d (6.2)
7	109.6, C		100.6, CH	5.86, s
8	159.9, C		139.3, C	
9	97.8, C		111.1, C	
10	136.4, C		163.5, C	
11	7.9, CH_3_	2.01, s	108.8, C	
12	41.7, CH_2_	2.47, m	162.4, C	
13	64.8, CH	4.02, m	108.6, CH	7.08, s
14	46.4, CH_2_	1.40, m	193.8, CH	10.08, s
15	62.7, CH	3.83, m	6.5, CH_3_	2.04, s
16	24.5, CH_3_	0.90, d (6.1)		
OH-6/10		10.84, s		12.87, s
OH-8/12		11.33, s		9.32, s
OH-13		4.72, d (5.6)		
OH-15		4.38, d (4.9)		

^a^ The ^1^H (400 M Hz) and ^13^C (100 M Hz); ^b^ The ^1^H (500 M Hz) and ^13^C (125 M Hz).

## Supplementary Materials

The following are available online at www.mdpi.com/1660-3397/15/1/2/s1: Figure S1: ^1^H NMR spectrum (400 MHz) of penicipyran A (**1**) in DMSO-*d*_6_, Figure S2: ^13^C NMR spectrum (100 MHz) of penicipyran A (**1**) in DMSO-*d*_6_, Figure S3: HMBC spectrum of penicipyran A (**1**) in DMSO-*d*_6_, Figure S4: HRESIMS spectrum of penicipyran A (**1**), Figure S5: IR spectrum of penicipyran A (**1**), Figure S6: UV spectrum of penicipyran A (**1**) in MeOH, Figure S7: ^1^H NMR spectrum (500 MHz) of penicipyran B (**2**) in acetone-*d*_6_, Figure S8: ^13^C NMR spectrum (125 MHz) of penicipyran B (**2**) in acetone-*d*_6_, Figure S9: HSQC spectrum of penicipyran B (**2**) in acetone-*d*_6_, Figure S10: HMBC spectrum of penicipyran B (**2**) in acetone-*d*_6_, Figure S11: HRESIMS spectrum of penicipyran B (**2**), Figure S12: IR spectrum of penicipyran B (**2**), Figure S13: UV spectrum of penicipyran B (**2**) in MeOH, Figure S14: ^1^H NMR spectrum (400 MHz) of penicipyran C (**3**) in DMSO-*d*_6_, Figure S15: ^13^C NMR spectrum (100 MHz) of penicipyran C (**3**) in DMSO-*d*_6_, Figure S16: DEPT spectrum of penicipyran C (**3**) in DMSO-*d*_6_, Figure S17: HSQC spectrum of penicipyran C (**3**) in DMSO-*d*_6_, Figure S18: HMBC spectrum of penicipyran C (**3**) in DMSO-*d*_6_, Figure S19: HRESIMS spectrum of penicipyran C (**3**), Figure S20: IR spectrum of penicipyran C (**3**), Figure S21: UV spectrum of penicipyran C (**3**) in MeOH, Figure S22: ^1^H NMR spectrum (400 MHz) of penicipyran D (**4**) in DMSO-*d*_6_, Figure S23: ^13^C NMR spectrum (100 MHz) of penicipyran D (**4**) in DMSO-*d*_6_, Figure S24: DEPT spectrum of penicipyran D (**4**) in DMSO-*d*_6_, Figure S25: ^1^H-^1^H COSY spectrum of penicipyran D (**4**) in DMSO-*d*_6_, Figure S26: HSQC spectrum of penicipyran D (**4**) in DMSO-*d*_6_, Figure S27: HMBC spectrum of penicipyran D (**4**) in DMSO-*d*_6_, Figure S28: ^1^H NMR spectrum (500 M Hz) of penicipyran D (**4**) in MeOH-*d*_6_, Figure S29: HRESIMS spectrum of penicipyran D (**4**), Figure S30: IR spectrum of penicipyran D (**4**), Figure S31: UV spectrum of penicipyran D (**4**) in MeOH, Figure S32: CD spectrum of penicipyran D (**4**) in MeOH, Figure S33: ^1^H NMR spectrum (400 M Hz) of **4a** in CDCl_3_, Figure S34: ^1^H NMR spectrum (400 M Hz) of **4b** in CDCl_3_, Figure S35: ^1^H NMR spectrum (500 MHz) of penicipyran E (**5**) in acetone-*d*_6_, Figure S36: ^13^C NMR spectrum (125 MHz) of penicipyran E (**5**) in acetone-*d*_6_, Figure S37: HSQC spectrum of penicipyran E (**5**) in acetone-*d*_6_, Figure S38: HMBC spectrum of penicipyran E (**5**) in acetone-*d*_6_, Figure S39: NOESY spectrum of penicipyran E (**5**) in acetone-*d*_6_, Figure S40: HRESIMS spectrum of penicipyran E (**5**), Figure S41: IR spectrum of penicipyran E (**5**), Figure S42: UV spectrum of penicipyran E (**5**) in MeOH, Figure S43: CD spectrum of penicipyran E (**5**) in MeOH.
