# Supplementary material for: Pyran Rings Containing Polyketides from Penicillium raistrickii
Source: Mar Drugs. 2016 Dec 23;15(1):2. doi: 10.3390/md15010002 (PMC5295222; doi:10.3390/md15010002)
Supplement: Supplementary file 1 [file marinedrugs-15-00002-s001.doc]

Supplementary Materials: Pyran Rings Containing Polyketides from *Penicillium raistrickii*

Li-Ying Ma, De-Sheng Liu, De-Guo Li, Yu-Ling Huang, Hui-Hui Kang, Chun-Hua Wang and
Wei-Zhong Liu


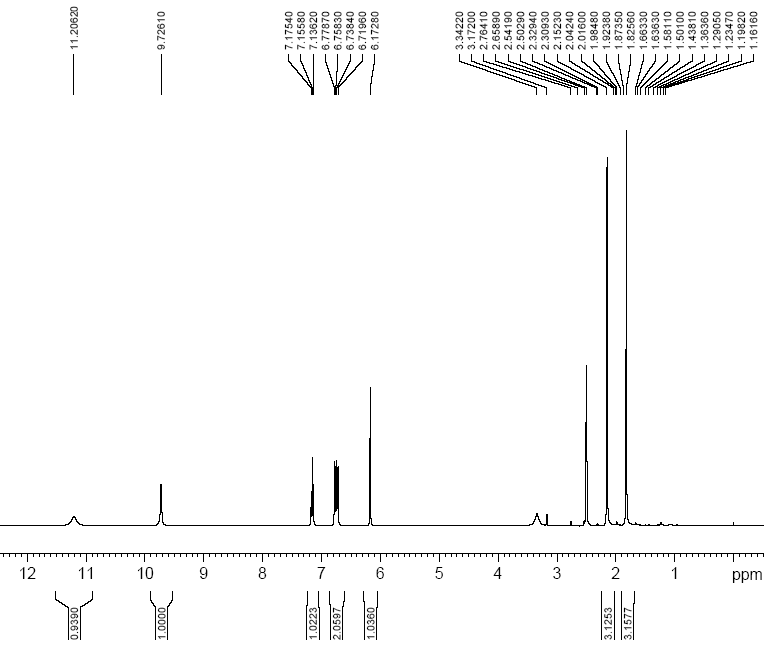


**Figure S1.** ^1^H NMR spectrum (400 MHz) of penicipyran A (**1**) in DMSO-*d*_6_.


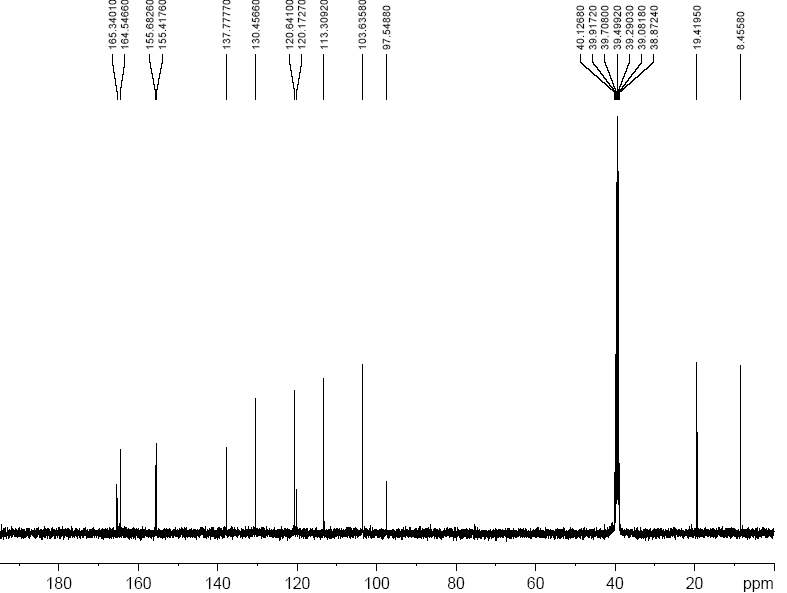


**Figure S2.** ^13^C NMR spectrum (100 MHz) of penicipyran A (**1**) in DMSO-*d*_6_.


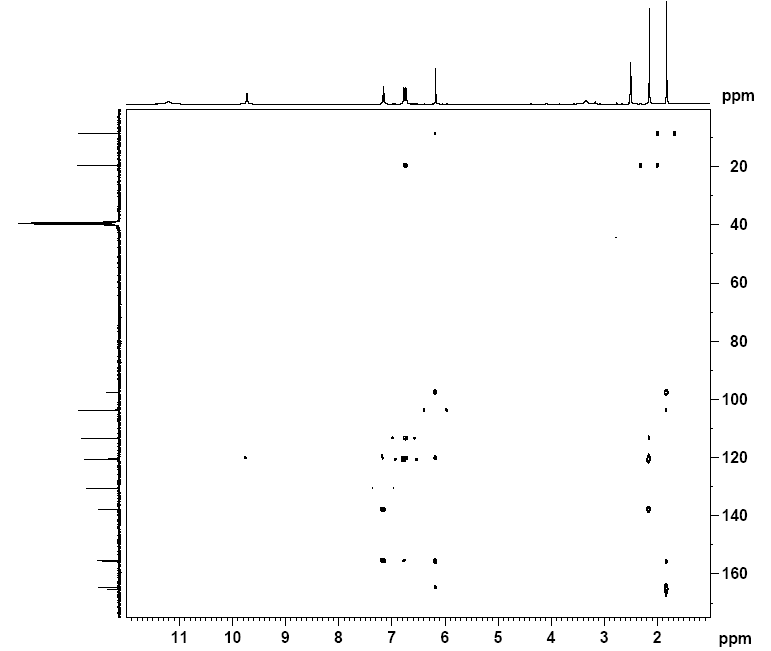


**Figure S3.** HMBC spectrum of penicipyran A (**1**) in DMSO-*d*_6_.


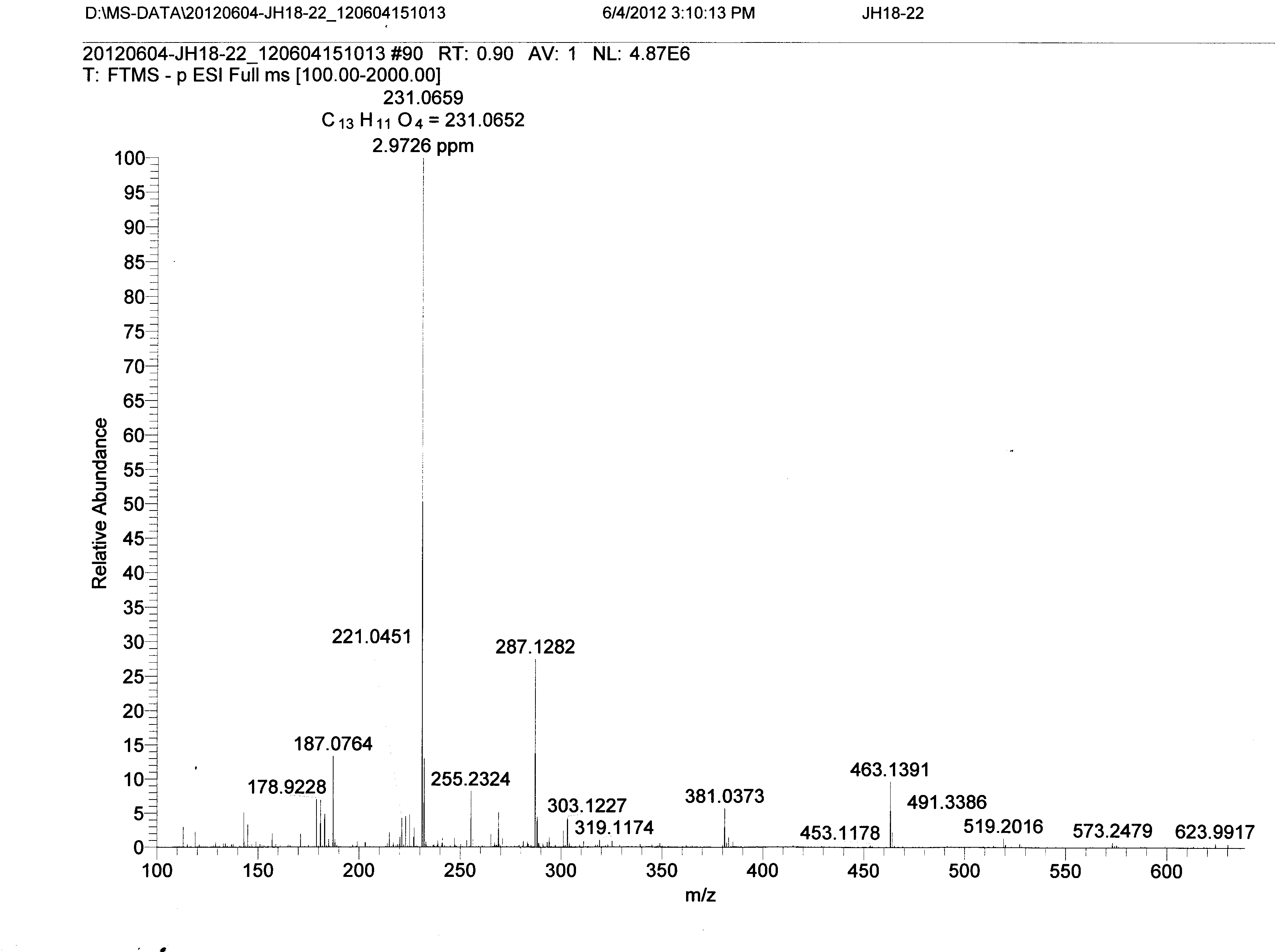


**Figure S4.** HRESIMS spectrum of penicipyran A (**1**).


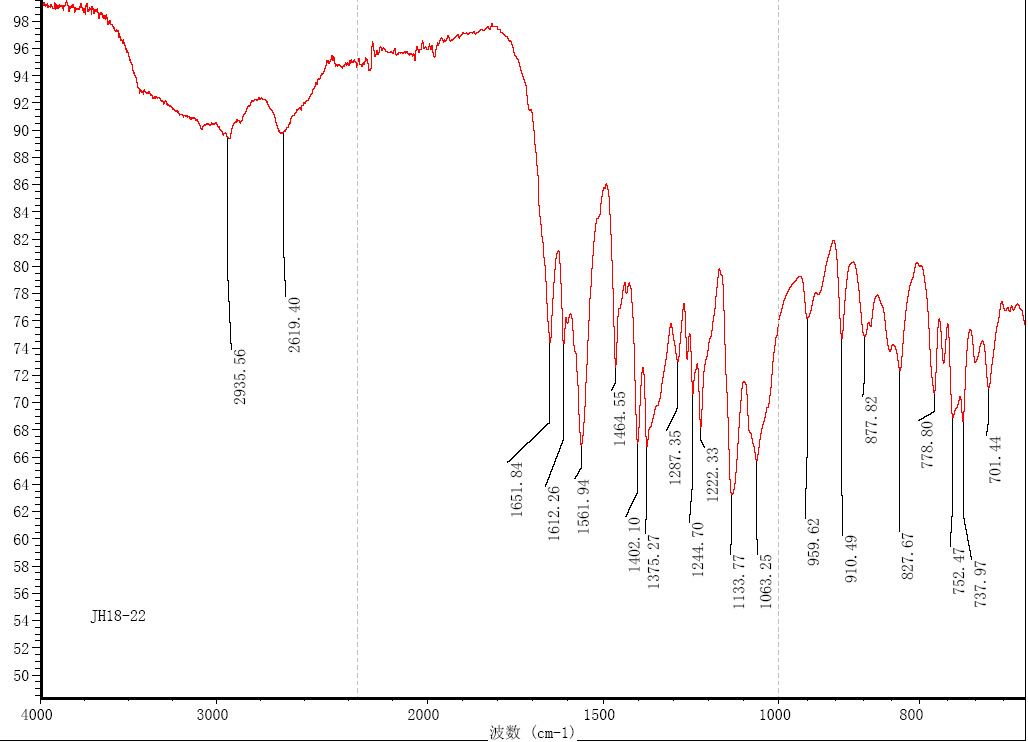


wavenumber (cm^-1^)

**Figure S5.** IR spectrum of penicipyran A (**1**).


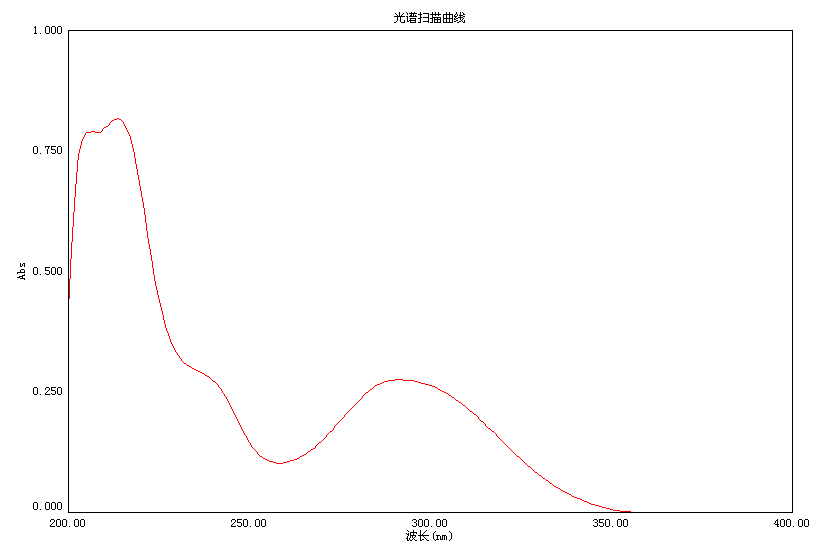


wavelength (nm)

**Figure S6.** UV spectrum of penicipyran A (**1**) in MeOH.


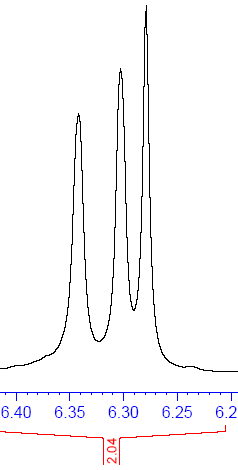

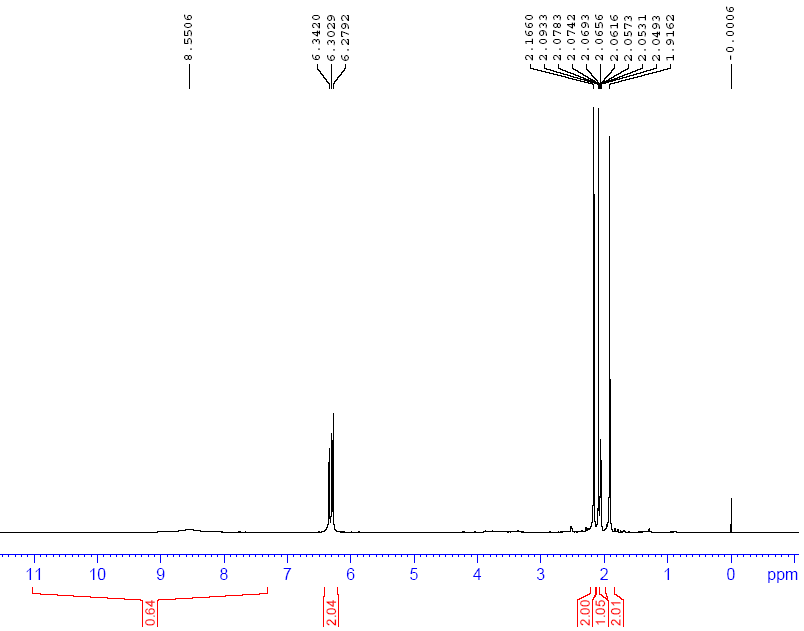


**Figure S7.** ^1^H NMR spectrum (500 MHz) of penicipyran B (**2**) in acetone-*d*_6_.


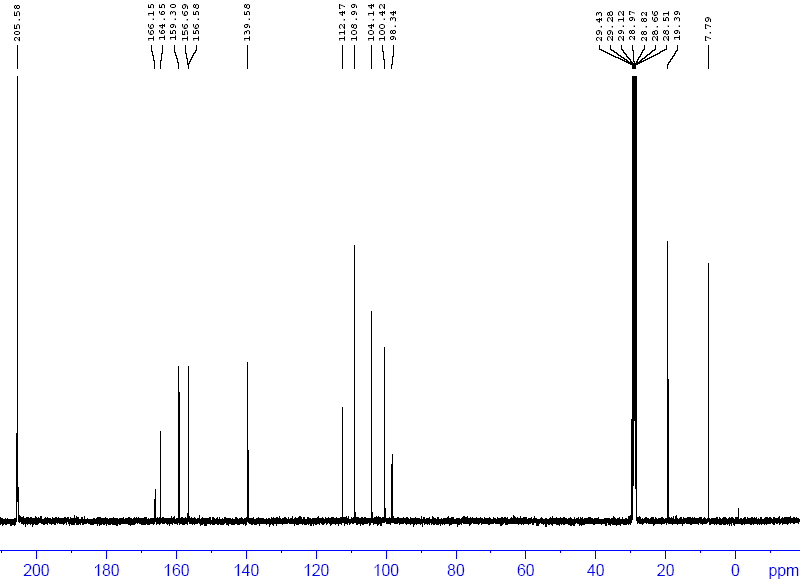


**Figure S8.** ^13^C NMR spectrum (125 MHz) of penicipyran B (**2**) in acetone-*d*_6_.


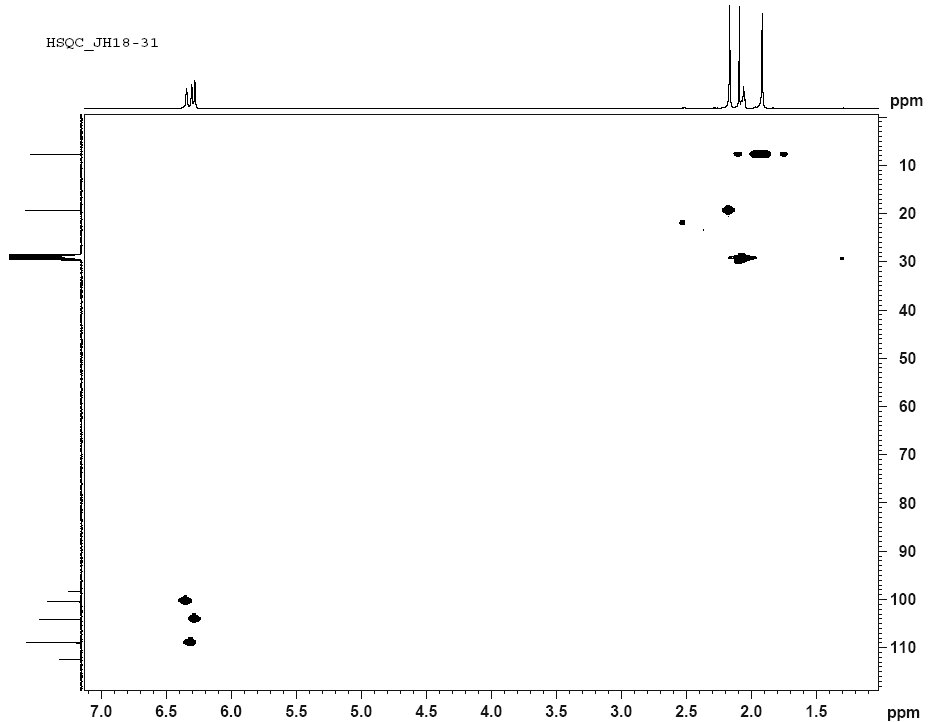


**Figure S9.** HSQC spectrum of penicipyran B (**2**) in acetone-*d*_6_.


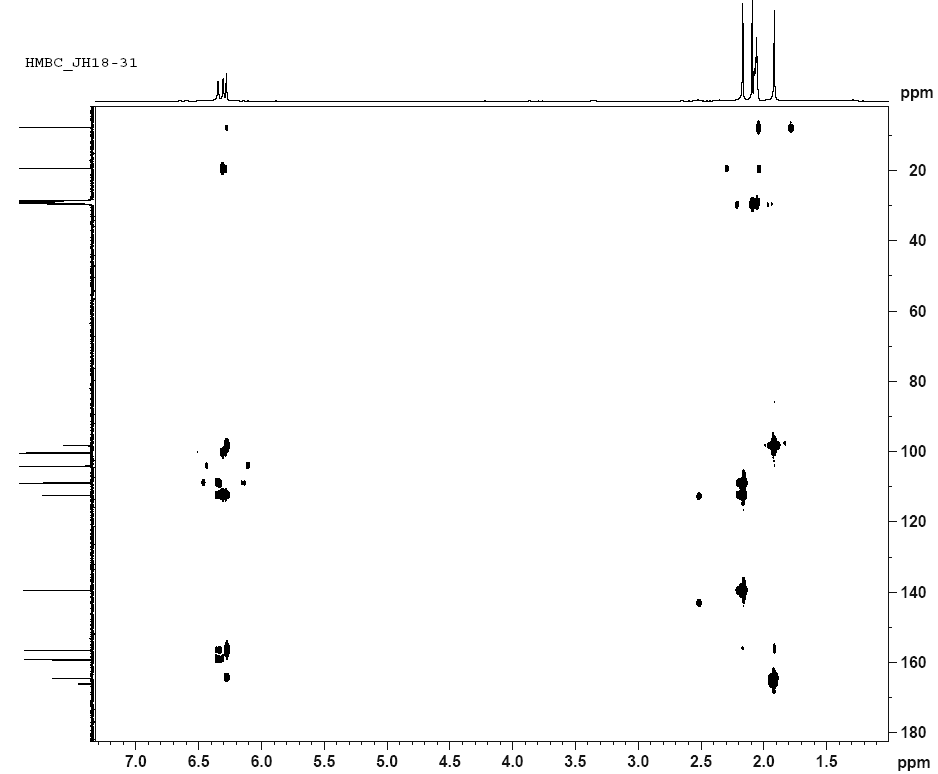


**Figure S10.** HMBC spectrum of penicipyran B (**2**) in acetone-*d*_6_.


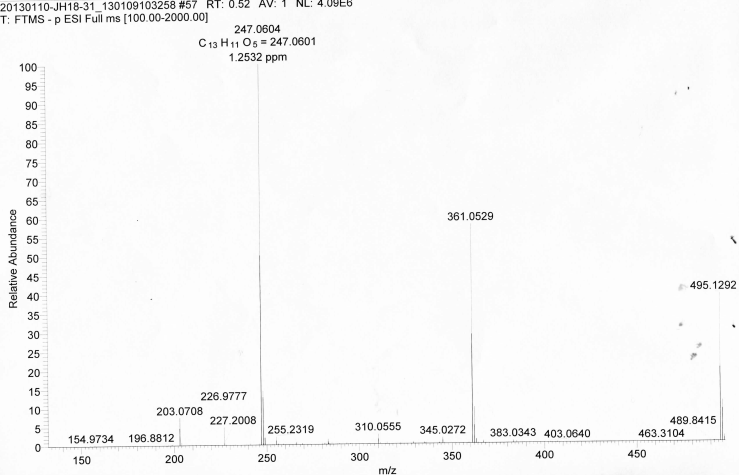


**Figure S11.** HRESIMS spectrum of penicipyran B (**2**).


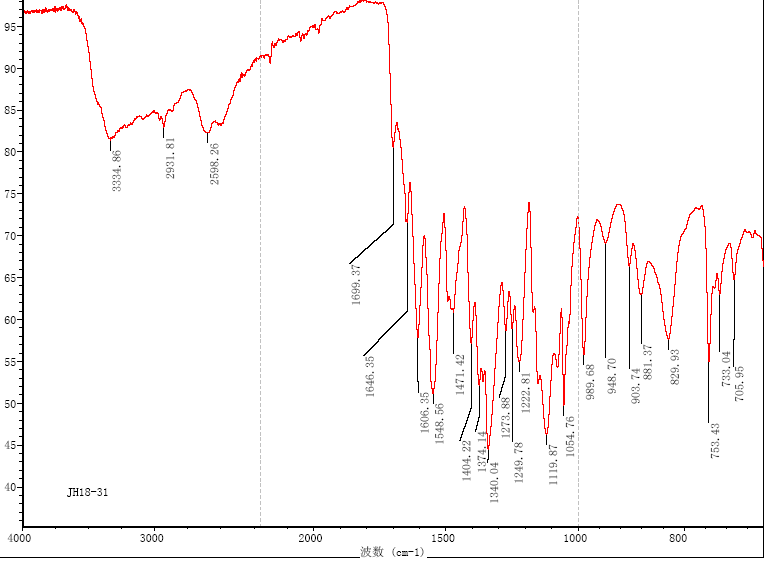


wavenumber (cm^-1^)

**Figure S12.** IR spectrum of penicipyran B (**2**).


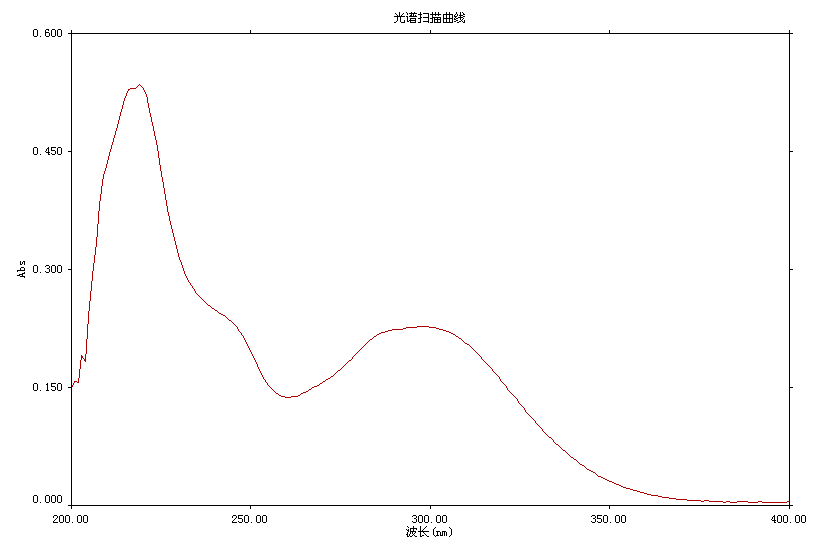


wavelength (nm)

**Figure S13.** UV spectrum of penicipyran B (**2**) in MeOH.


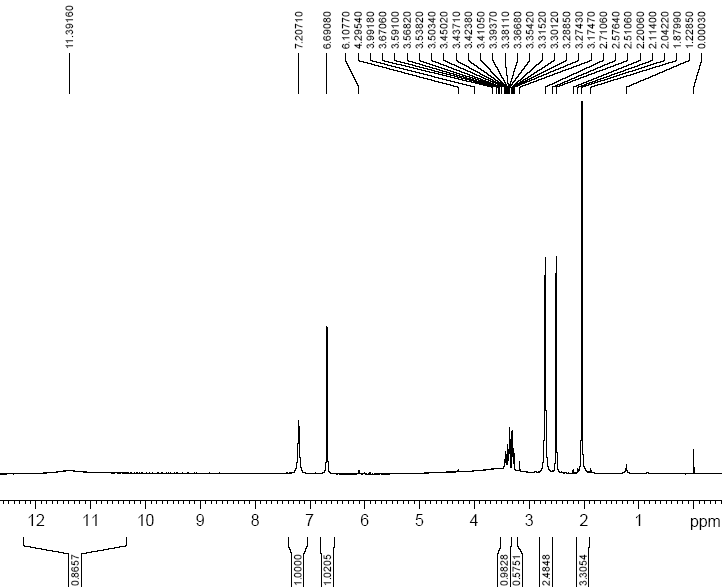


glycerol

trimethylamineol

**Figure S14.** ^1^H NMR spectrum (400 MHz) of penicipyran C (**3**) in DMSO-*d*_6_.


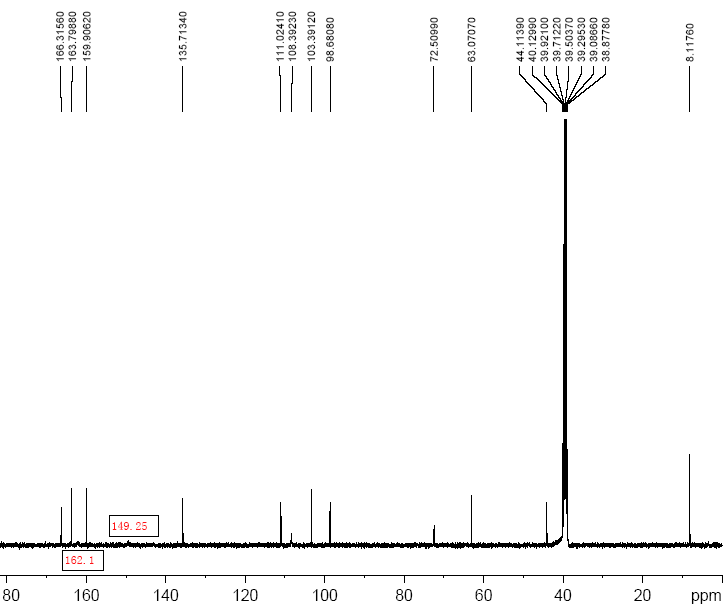


glycerol

trimethylamineol

**Figure S15.** ^13^C NMR spectrum (100 MHz) of penicipyran C (**3**) in DMSO-*d*_6_.


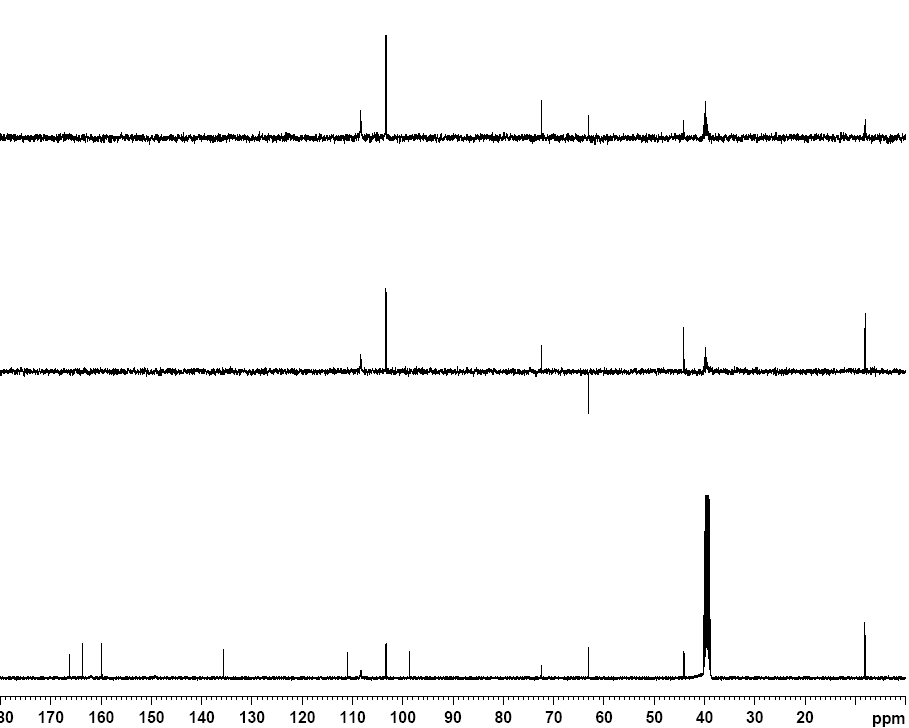


**Figure S16.** DEPT spectrum of penicipyran C (**3**) in DMSO-*d*_6_.


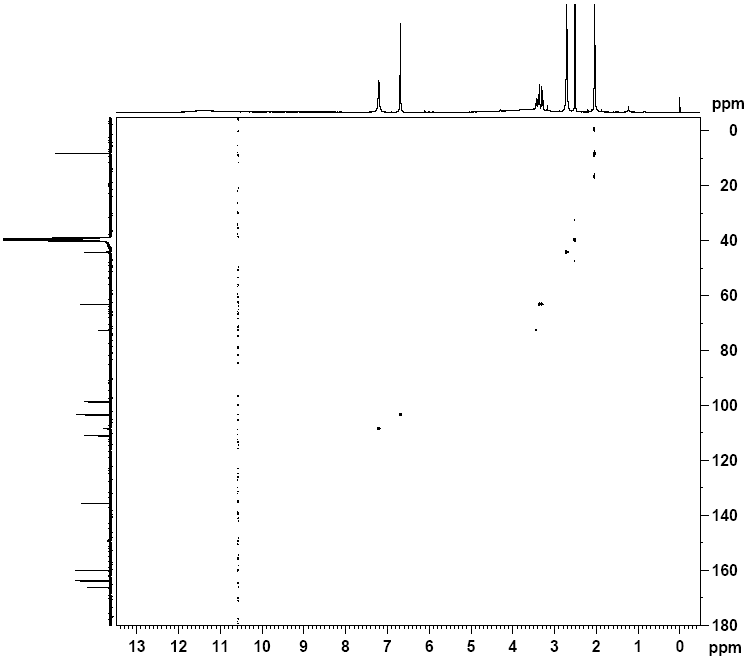


**Figure S17.** HSQC spectrum of penicipyran C (**3**) in DMSO-*d*_6_.


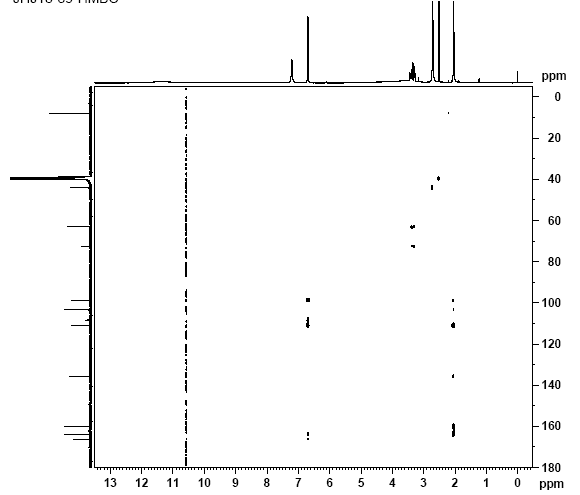


**Figure S18.** HMBC spectrum of penicipyran C (**3**) in DMSO-*d*_6_.


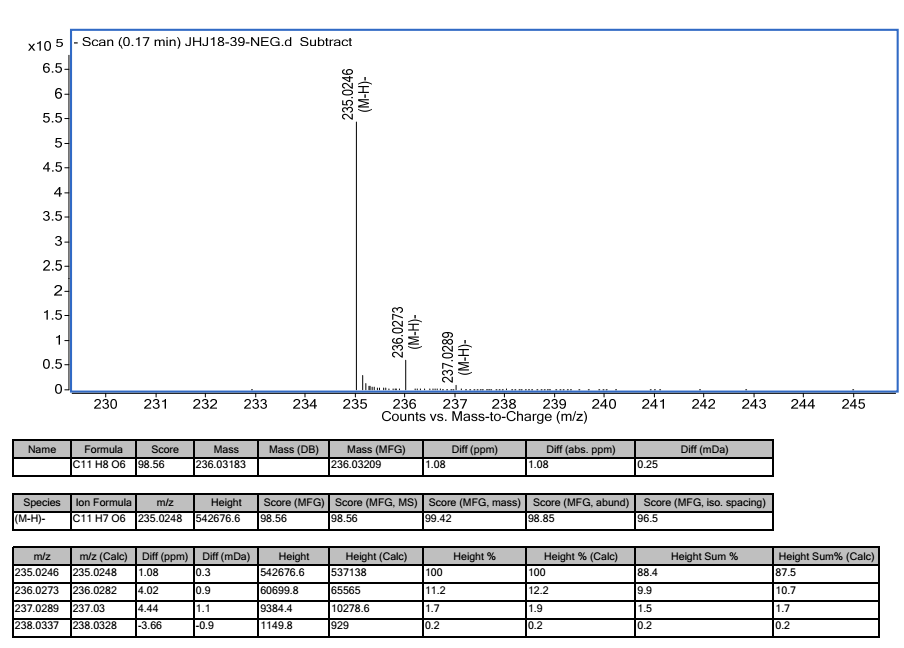


**Figure S19.** HRESIMS spectrum of penicipyran C (**3**).


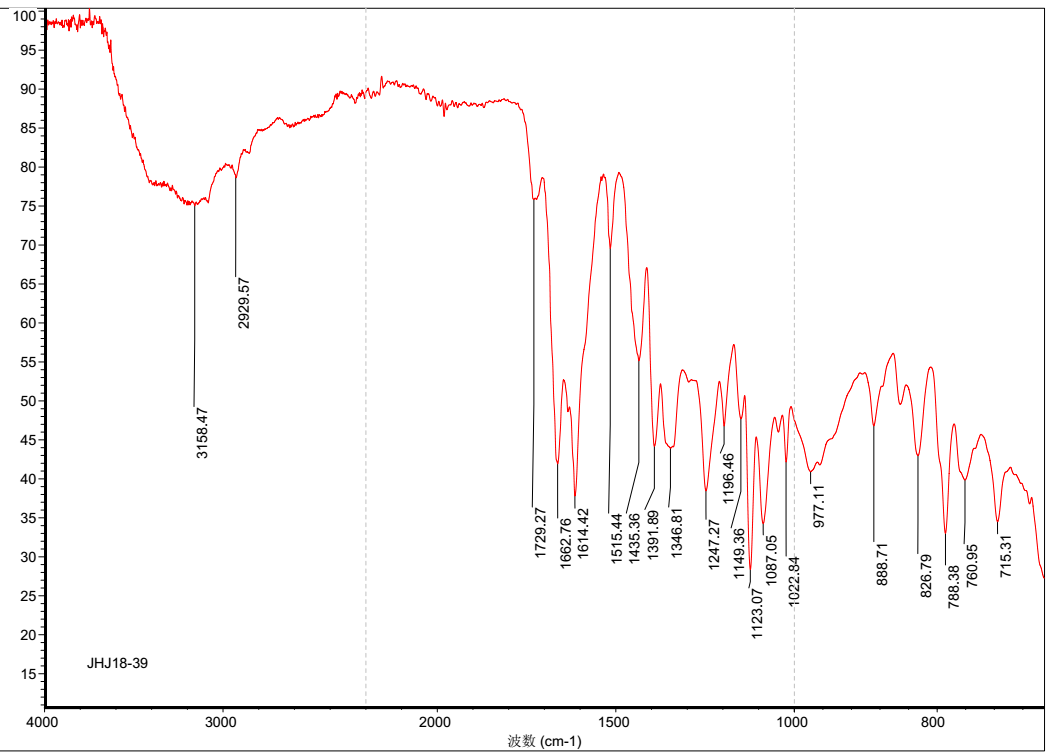


wavenumber (cm^-1^)

**Figure S20.** IR spectrum of penicipyran C (**3**).


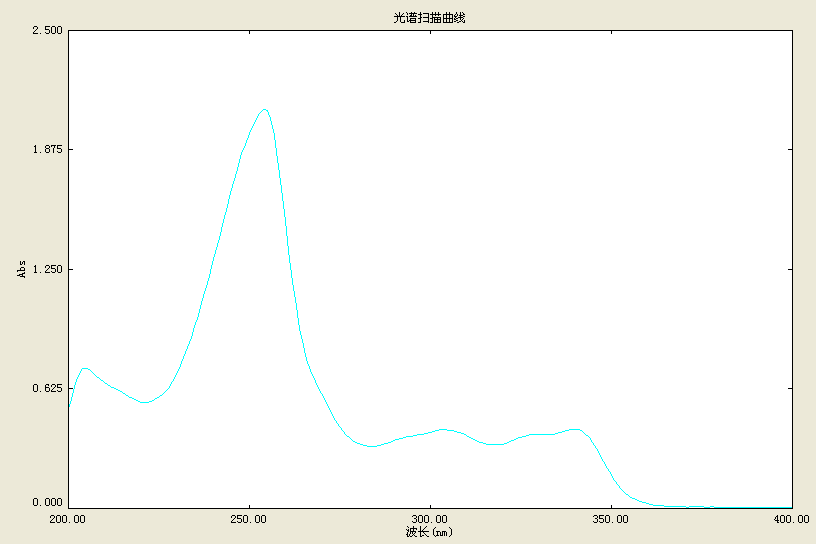


wavelength (nm)

**Figure S21.** UV spectrum of penicipyran C (**3**) in MeOH.


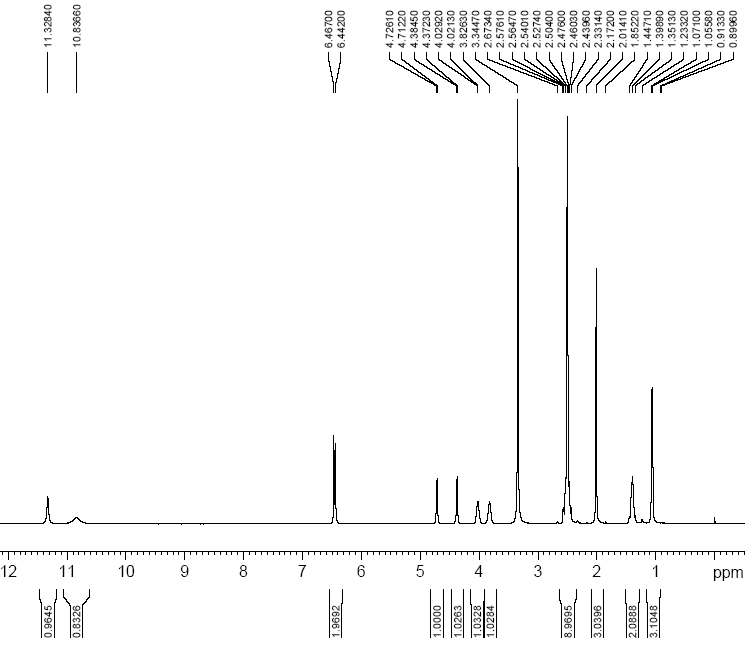


**Figure S22.** ^1^H NMR spectrum (400 MHz) of penicipyran D (**4**) in DMSO-*d*_6_.


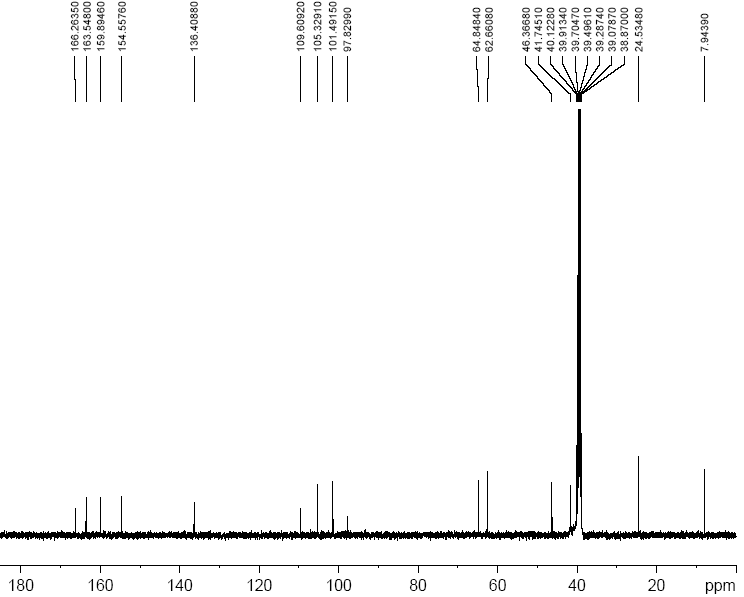


**Figure S23.** ^13^C NMR spectrum (100 MHz) of penicipyran D (**4**) in DMSO-*d*_6_


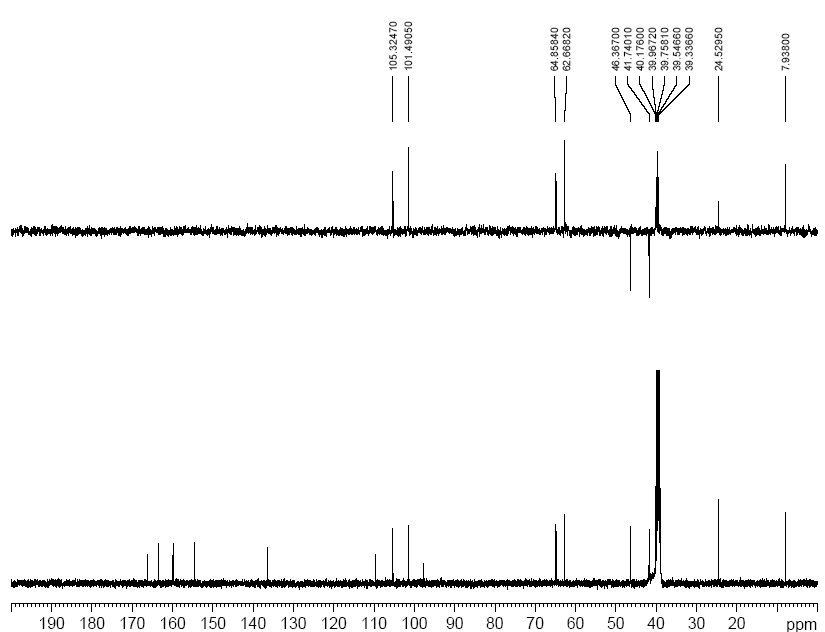


**Figure S24.** DEPT spectrum of penicipyran D (**4**) in DMSO-*d*_6_.


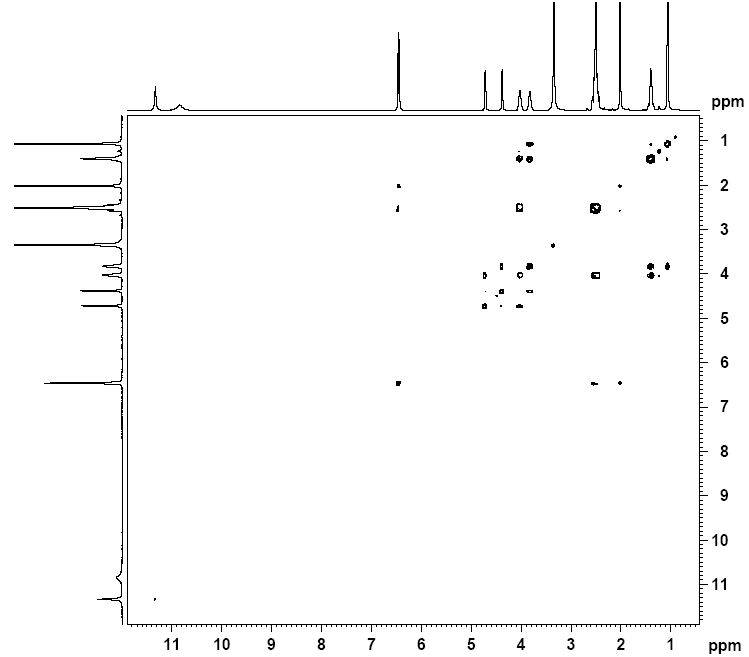


**Figure S25.** ^1^H-^1^H COSY spectrum of penicipyran D (**4**) in DMSO-*d*_6_.


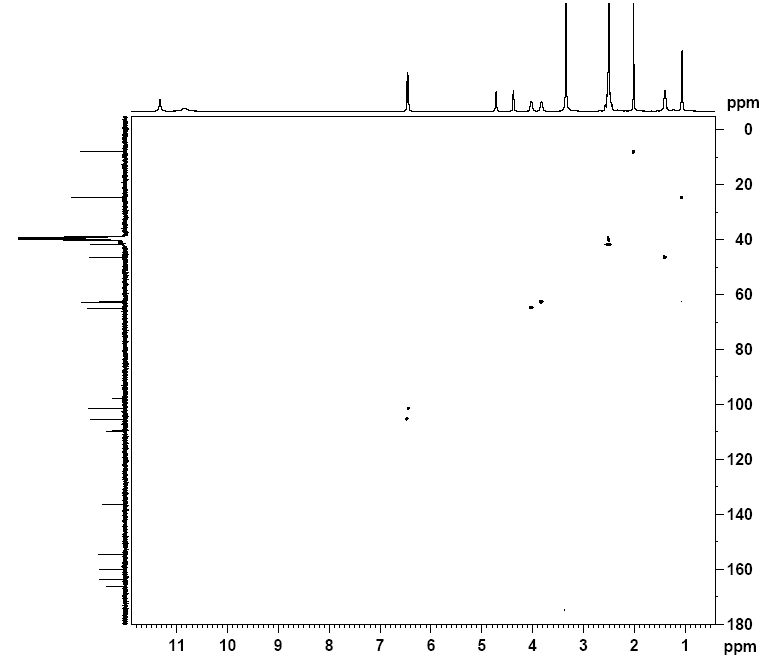


**Figure S26.** HSQC spectrum of penicipyran D (**4**) in DMSO-*d*_6_.


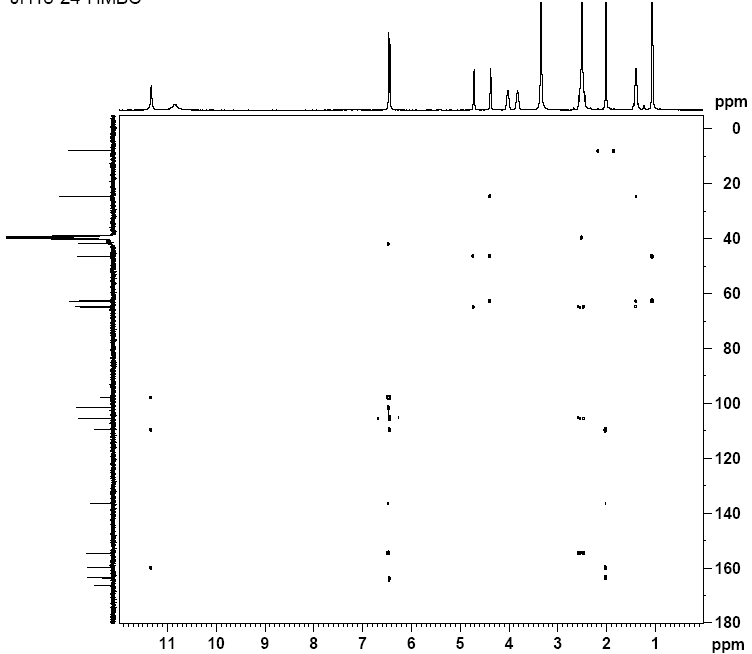


**Figure S27.** HMBC spectrum of penicipyran D (**4**) in DMSO-*d*_6_.


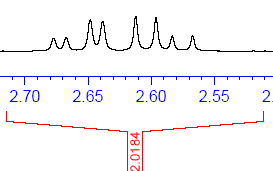

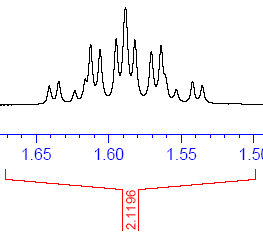

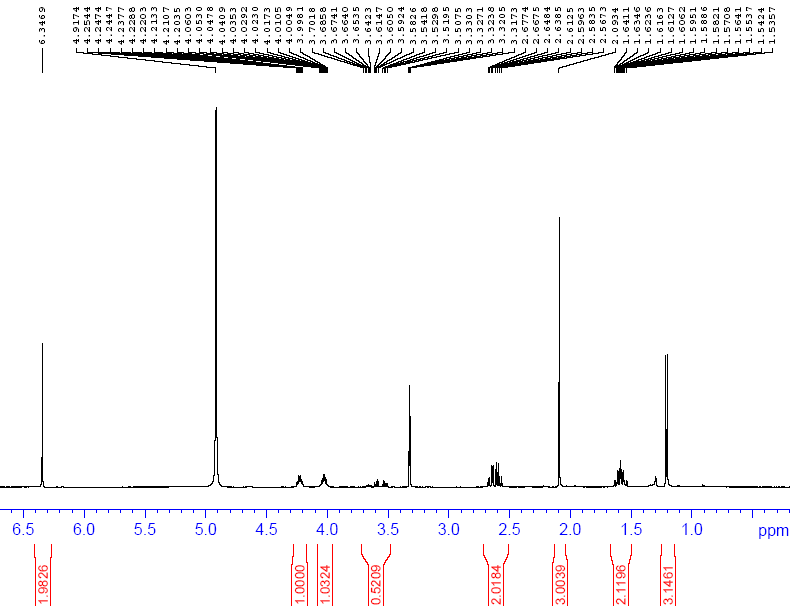


H-14

H-12

**Figure S28.** ^1^H NMR spectrum (500 M Hz) of penicipyran D (**4**) in MeOH-*d*_6_.


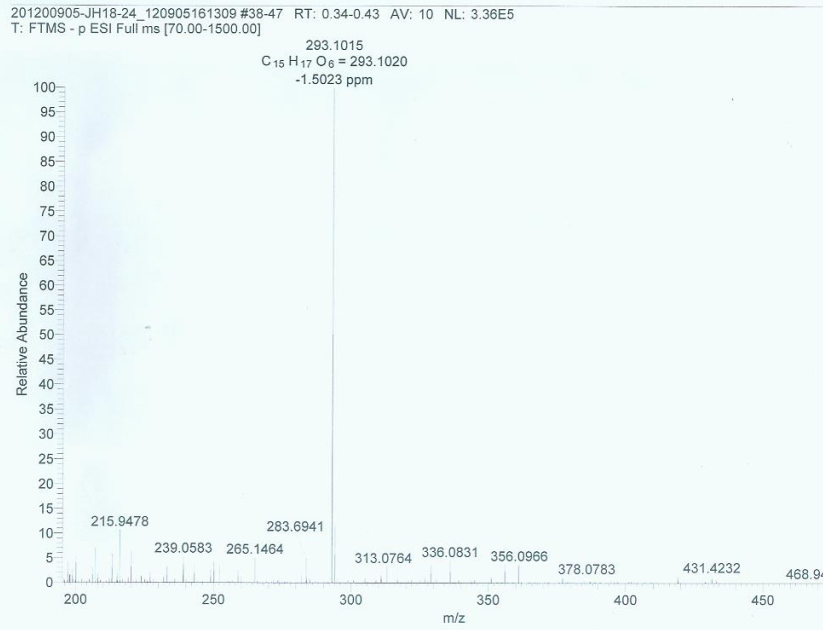


**Figure S29.** HRESIMS spectrum of penicipyran D (**4**).


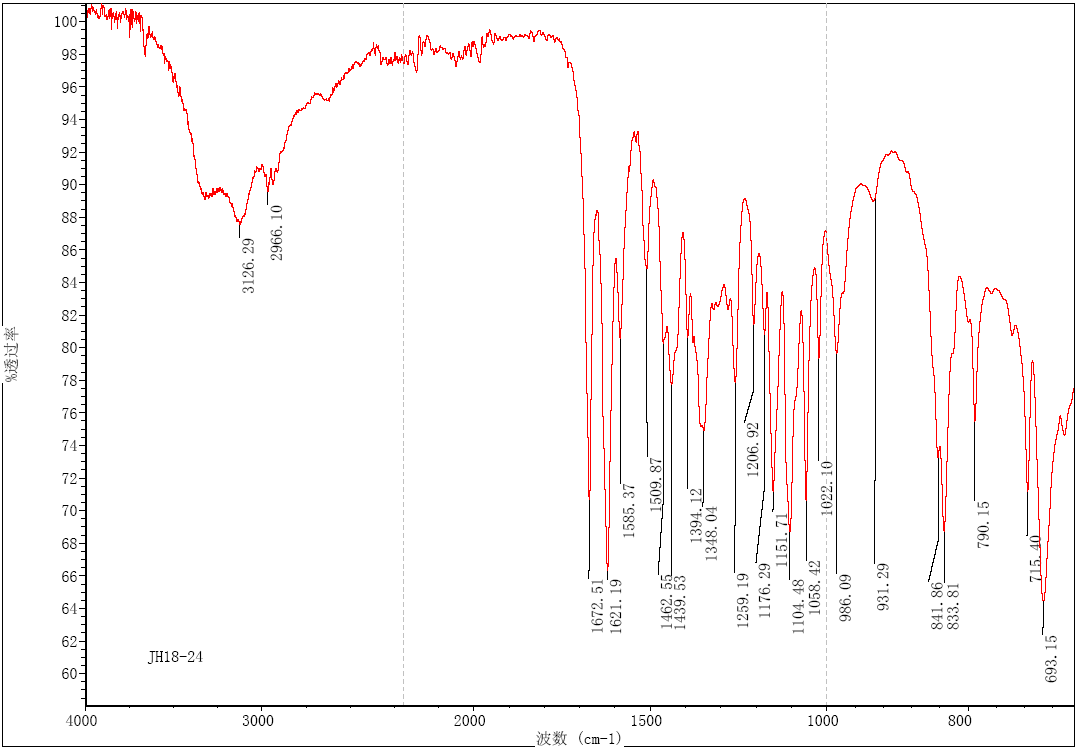


wavenumber (cm^-1^)

**Figure S30.** IR spectrum of penicipyran D (**4**).


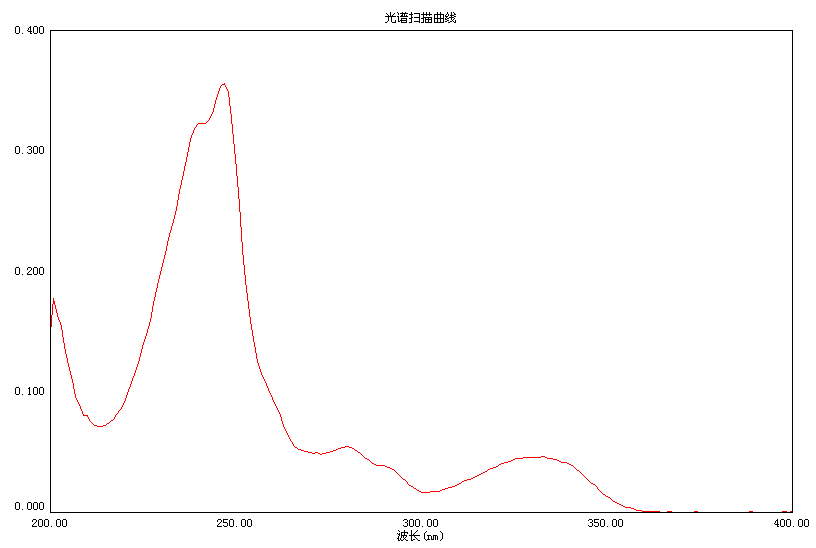


wavelength (nm)

**Figure S31.** UV spectrum of penicipyran D (**4**) in MeOH.


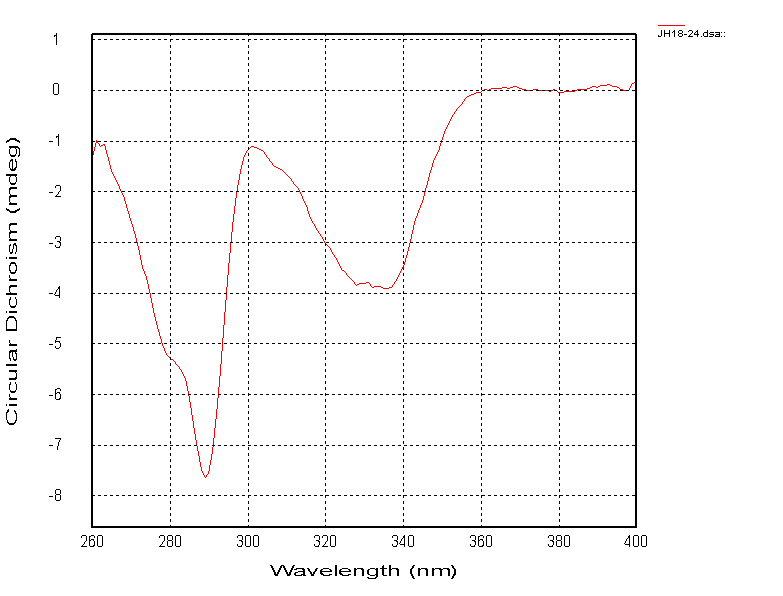


**Figure S32.** CD spectrum of penicipyran D (**4**) in MeOH.


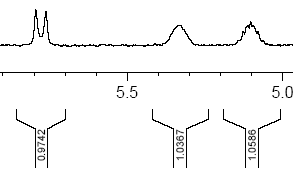

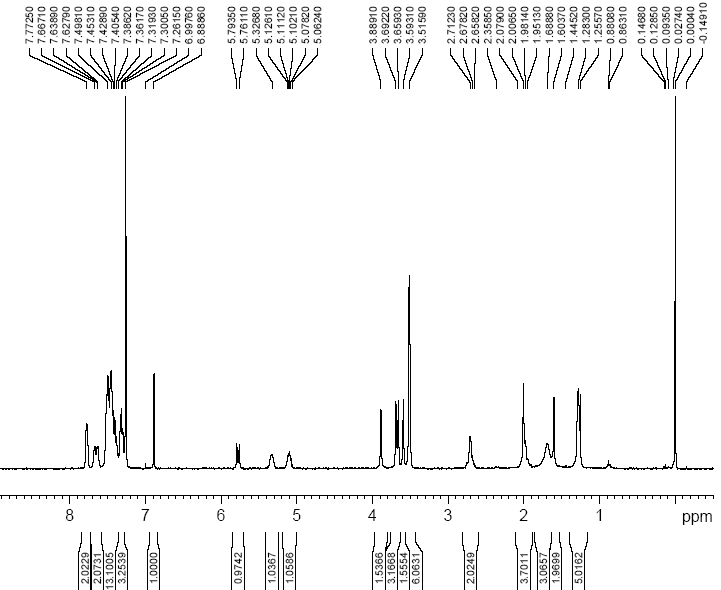


H-13

H-15

**Figure S33.** ^1^H NMR spectrum (400 M Hz) of **4a** in CDCl_3_.


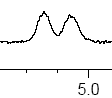

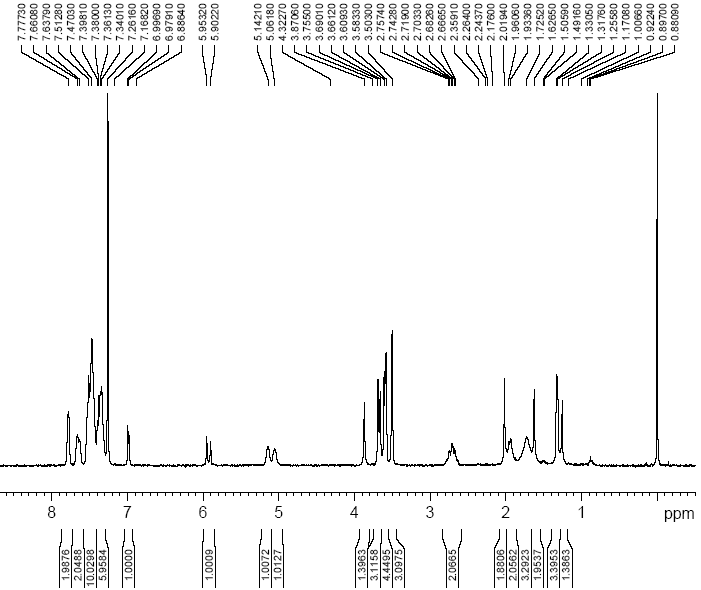


H-13

H-15

**Figure S34.** ^1^H NMR spectrum (400 M Hz) of **4b** in CDCl_3_.


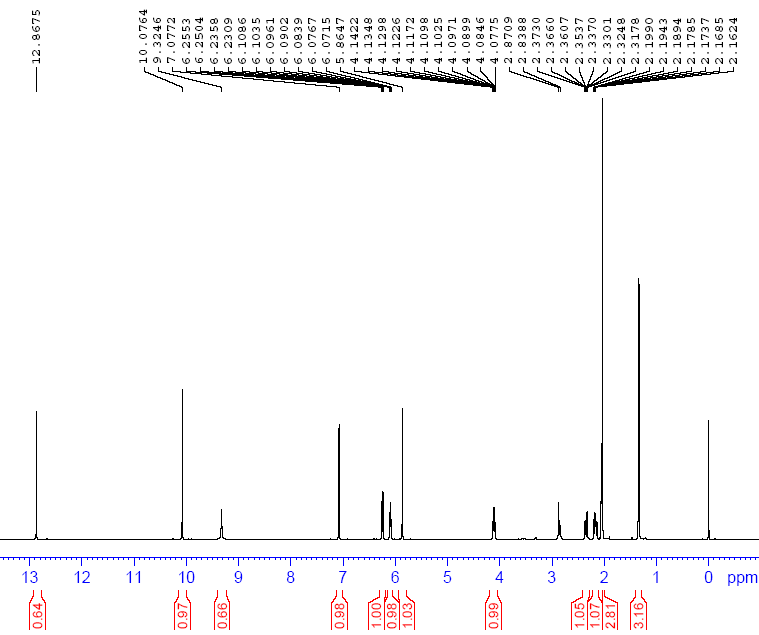


**Figure S35.** ^1^H NMR spectrum (500 MHz) of penicipyran E (**5**) in acetone-*d*_6_.


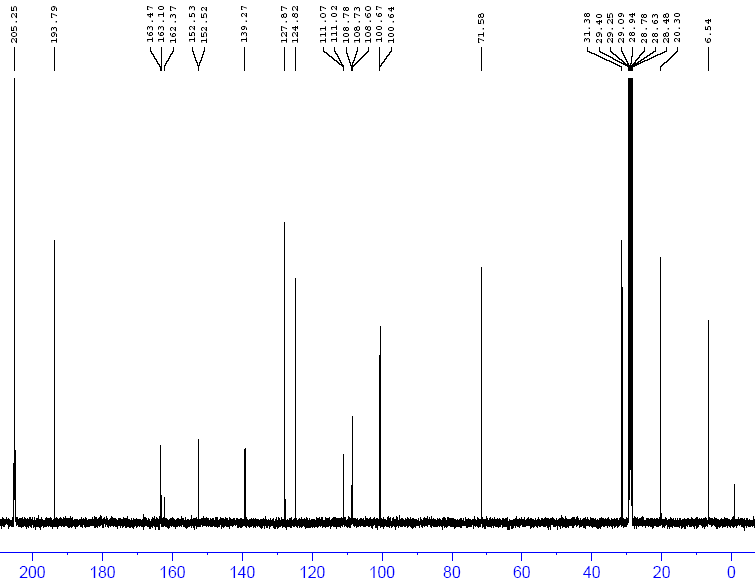


**Figure S36.** ^13^C NMR spectrum (125 MHz) of penicipyran E (**5**) in acetone-*d*_6_.


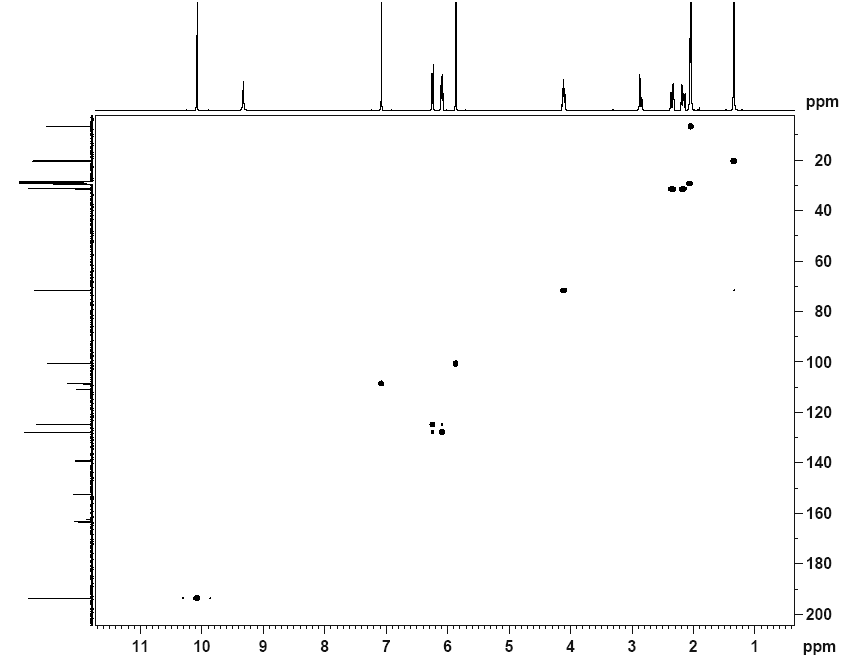


**Figure S37.** HSQC spectrum of penicipyran E (**5**) in acetone-*d*_6_.


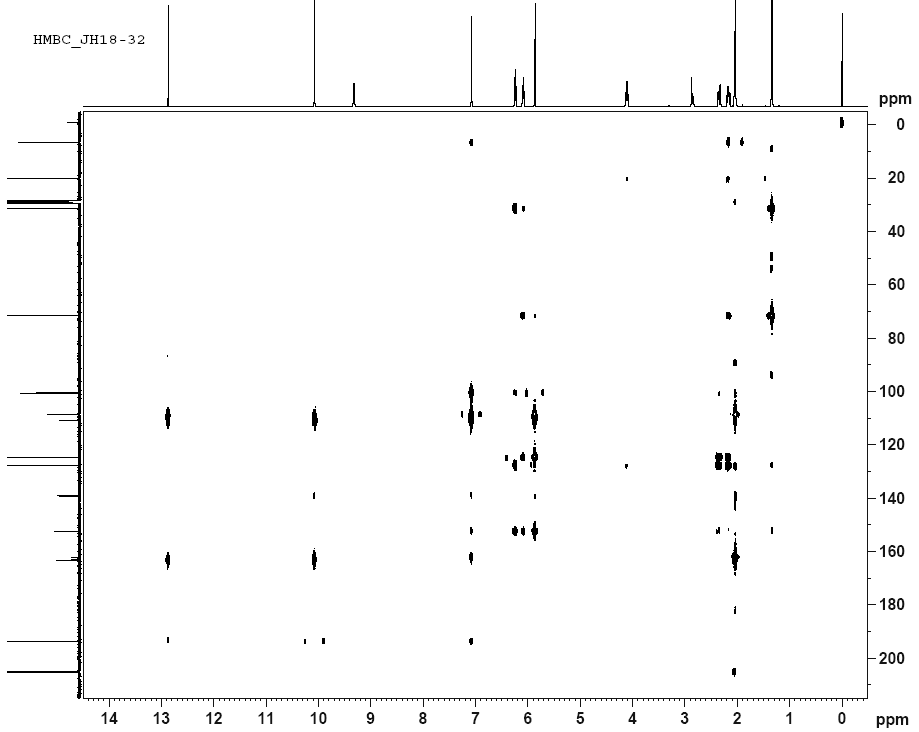


**Figure S38.** HMBC spectrum of penicipyran E (**5**) in acetone-*d*_6_.


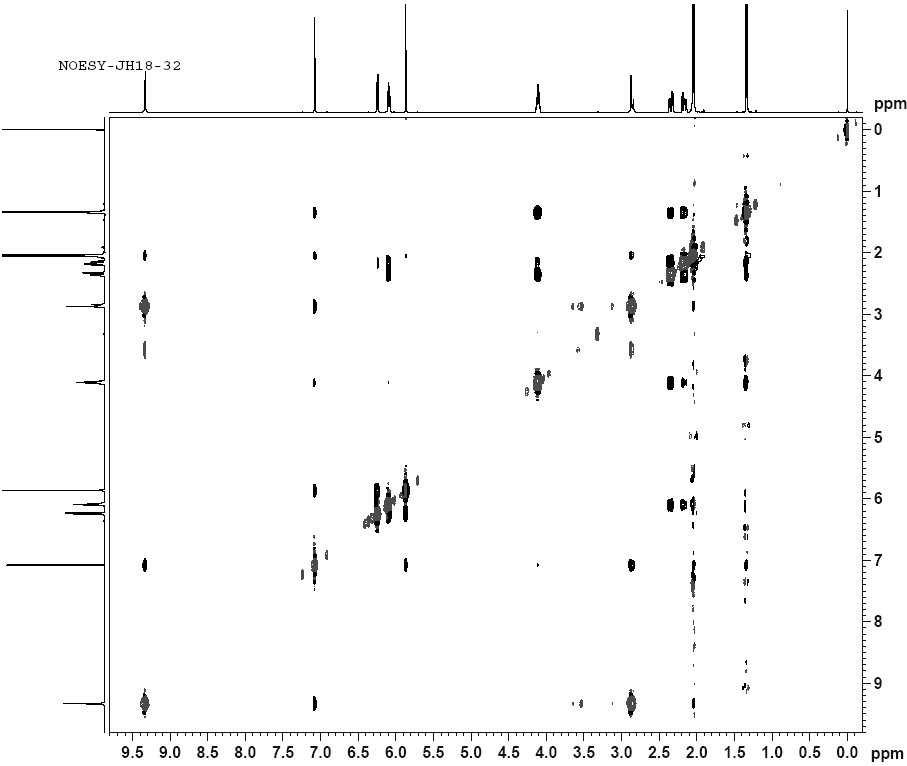


**Figure S39.** NOESY spectrum of penicipyran E (**5**) in acetone-*d*_6_.


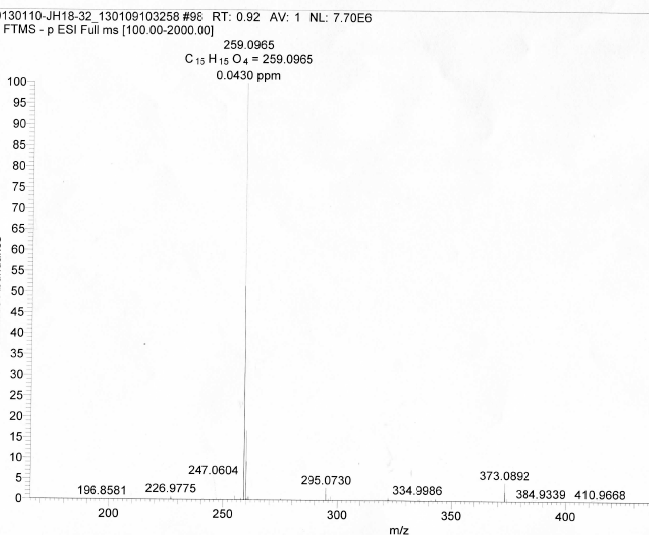


**Figure S40.** HRESIMS spectrum of penicipyran E (**5**).


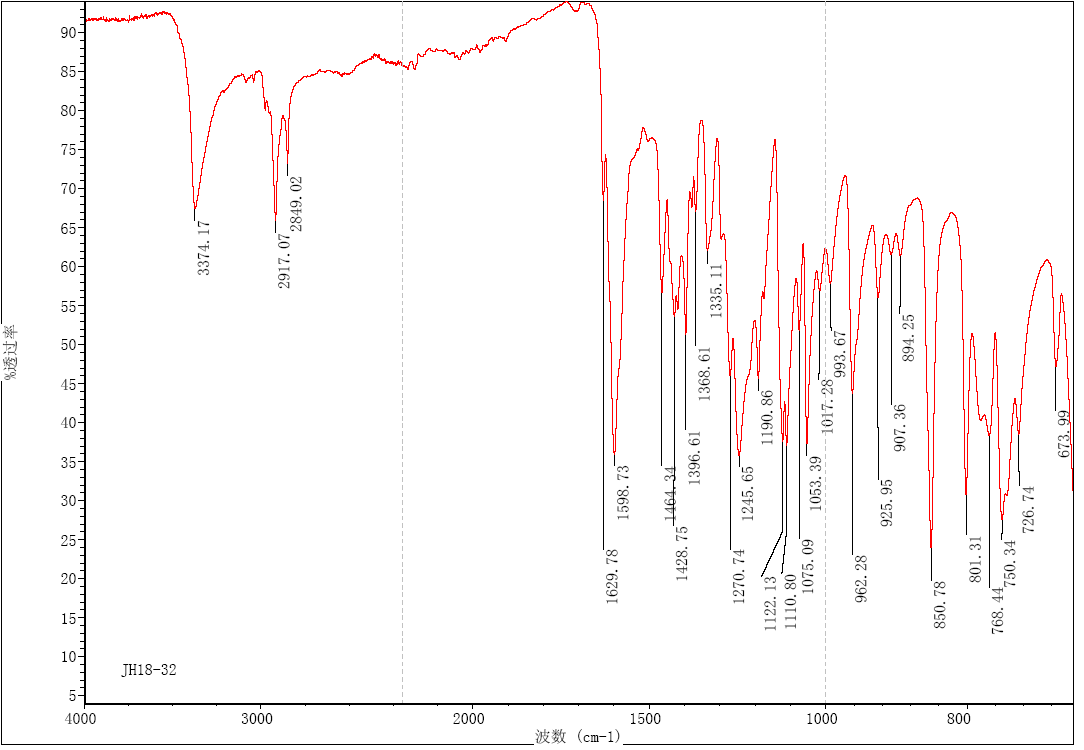


wavenumber (cm^-1^)

**Figure S41.** IR spectrum of penicipyran E (**5**).


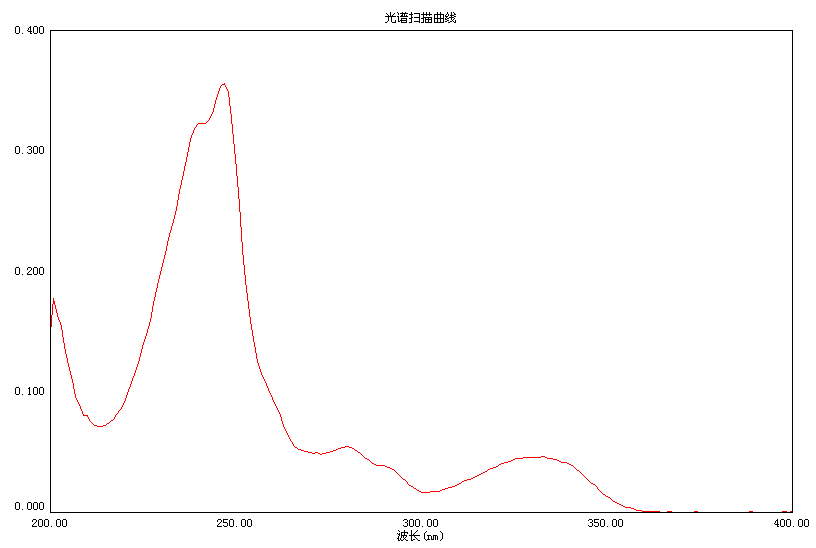


wavelength (nm)

**Figure S42.** UV spectrum of penicipyran E (**5**) in MeOH.


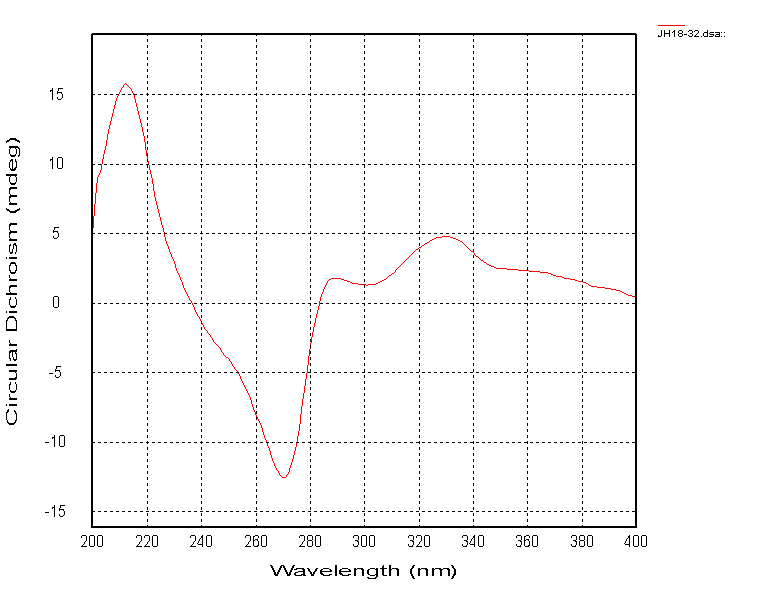


**Figure S43.** CD spectrum of penicipyran E (**5**) in MeOH.
